# Rhizobial motility preference in root colonization of *Medicago truncatula*

**DOI:** 10.1111/nph.70897

**Published:** 2026-01-15

**Authors:** Anaïs Delers, Anne Bennion, Ambre Guillory, Lisa Frances, Elizaveta Krol, Fanny Bonnafous, Laurena Medioni, Javier Serrania, Rémi Peyraud, Joëlle Fournier, Fernanda de Carvalho‐Niebel, Anke Becker

**Affiliations:** ^1^ LIPME, INRAE, CNRS Université de Toulouse 31320 Castanet‐Tolosan France; ^2^ Center for Synthetic Microbiology Philipps‐Universität Marburg 35032 Marburg Germany; ^3^ Department of Biology Philipps‐Universität Marburg 35032 Marburg Germany; ^4^ iMEAN 31077 Toulouse France; ^5^ LRSV, Université de Toulouse, CNRS, UPS, Toulouse INP 31320 Castanet‐Tolosan France

**Keywords:** flagella, infection thread, *Medicago truncatula*, motility, rhizobactin, rhizobia

## Abstract

- Tunnel‐like infection thread (IT) structures support root colonization by symbiotic nitrogen‐fixing rhizobia bacteria in most legume species. These tip‐grown structures are key to directing rhizobia from root hairs to developing nodules, where they are hosted to fix nitrogen. Rhizobia likely progress inside ITs by combining growth and motility by modes not yet defined.
- Here, we tackled this question by combining mathematical modeling, live cell imaging, and bacterial mutant phenotyping in *Medicago truncatula*.
- Modeling the motion of fluorescently‐labeled *Sinorhizobium meliloti* inside root hair IT compartments estimated slow movement (2–6 μm h^−1^), compatible with passive rather than active motility. Consistent with this model, flagella‐less *S. meliloti* mutants were impaired in active swimming motility *in vitro*, yet could colonize host roots and nodules *in planta*. By contrast, mutation in the rhizobactin 1021 siderophore *rhbE* biosynthesis gene affected surface motility *in vitro* and host root and nodule colonization. This mutation also promoted the formation of branched ITs in root hairs, which ultimately resulted in impaired nodule development and infection.
- In line with the slow motion of *S. meliloti* inside ITs estimated by modeling, our findings suggest that rhizobia favor flagella‐independent surface translocation to reach developing nodules in *M. truncatula*.

## Introduction

Symbiotic relationships with soil microorganisms can help plants access the nutrients they need for growth. Certain angiosperm species in the nitrogen‐fixing clade evolved the ability to obtain nitrogen through symbiosis with bacteria, which they host intracellularly in specialized organs called root nodules (Huisman & Geurts, 2020). These interactions have been well‐studied in legume plants, notably in model species such as *Medicago truncatula*, which hosts the rhizobia symbiont *Sinorhizobium meliloti*. Establishing this interaction requires precise molecular exchanges between partners (Krönauer & Radutoiu, 2021) before bacteria can colonize their host, which occurs in most cases via a tunnel‐like apoplastic compartment called the infection thread (IT) (Gage, 2004).

ITs initiate in root hairs and proceed through well‐defined stages (reviewed in de Carvalho‐Niebel *et al*., 2024). Root hairs curl around Nod factor‐producing rhizobia (Esseling *et al*., 2003) and enclose them in a radially expanding infection chamber where they proliferate until polarized secretion creates the tip‐growing IT tubular structure (Fournier *et al*., 2015). ITs are sequentially reinitiated in successive cell layers to guide rhizobia transcellularly from root hairs to the developing nodule primordium, where they are released, endocytosed, and differentiated into N‐fixing bacteroids (Yang *et al*., 2022).

The successful formation and progression of ITs within plant cells depends on the plant's specific perception or controlled degradation of rhizobial Nod factors or exopolysaccharide signals (Kawaharada *et al*., 2017; Malolepszy *et al*., 2018). The plant host also triggers a series of cellular events (reviewed in de Carvalho‐Niebel *et al*., 2024) to create the optimal IT apoplastic environment for rhizobia colonization. Inside the IT space, rhizobia progress in a sparse, single‐file arrangement, slightly behind the IT tip that extends in a cytoplasmic bridge connected with the migrating nucleus (Fournier *et al*., 2008; Guillory *et al*., 2024). It has been proposed that rhizobia progress in the IT environment by combining cell proliferation and collective movement, though the form of motility they actually use is unknown.

Bacteria can adopt different types of movement to translocate. These can be categorized as swimming motility in aqueous solutions or surface‐associated motility in solid environments. While swimming motility depends on flagellar rotation, surface motility relies on different mechanisms, with the most prominent modes being twitching, gliding, swarming, and sliding (reviewed by Wadhwa & Berg, 2022). Twitching is mediated by type IV pili, which extend, adhere to a surface and retract to pull the cell forward (Maier & Wong, 2015; Craig *et al*., 2019). Gliding involves focal adhesion complexes that attach to a surface and move across the length of the cell (McBride, 2001; Kearns, 2011). Swarming is a collective movement driven by flagella that occurs at high cell density in a thin layer of fluid on top of a surface, generated through the secretion of osmolytes or surfactants (Kearns, 2011; Wadhwa & Berg, 2022). Sliding is a form of passive motility independent of flagella, pili, and adhesion complexes that relies instead on the pressure exerted from cell proliferation, aided by reduced friction through the release of surfactants (Holscher & Kovacs, 2017).

*Sinorhizobium meliloti* is capable of swimming (Gotz & Schmitt, 1987) and moving on surfaces via swarming and sliding (Soto *et al*., 2002; Nogales *et al*., 2012), but no formal evidence exists for gliding or twitching motility in *S. meliloti* (Zatakia *et al*., 2014; Wadhwa & Berg, 2022), as genes essential for gliding or production of Type IVa pili, involved in twitching motility, are not present in its genome. Swimming motility by individual *S. meliloti* is enabled by the rotation of peritrichous flagella (Gotz & Schmitt, 1987), which also contribute to the collective swarming movement of *S. meliloti* across solid surfaces (Nogales *et al*., 2012). However, *S. meliloti* can also spread on surfaces independent of flagella, which has been attributed to sliding (Nogales *et al*., 2012).

In *S. meliloti*, the assembly of flagellar functional parts – the filament, the basal body with the motor for filament propelling, and the hook joining the two parts – is governed by three class‐hierarchically regulated genes that cluster in a contiguous 45 kb chromosomal region (Sourjik *et al*., 1998, 2000). Flagellar biosynthesis in *S. meliloti* is also regulated by the ExpR/Sin quorum‐sensing system, which can downregulate the expression of flagellar biosynthesis genes at high population density (Hoang *et al*., 2004). This quorum‐sensing system also regulates the production of exopolysaccharides (Hoang *et al*., 2004), including symbiotically active EPS II (galactoglucan), which promotes an unusual surface motility mostly driven by entropic forces (i.e. osmotic flow and depletion attraction) referred to as surfing (Dilanji *et al*., 2014). *Sinorhizobium meliloti* strains 1021 and 2011, frequently employed for symbiotic studies in *M. truncatula*, are disrupted in *expR* (Pellock *et al*., 2002). Although EPS II biosynthesis is reduced in these strains, surface motility is still observed, albeit dependent on the biosynthesis of the siderophore rhizobactin (Rhb) 1021 (Nogales *et al*., 2010, 2012). Thus, Rhb1021 may act as a surfactant to facilitate surface motility.

Siderophores are high‐affinity iron chelators secreted by many organisms, including bacteria (Timofeeva *et al*., 2022). Rhb1021 is composed of a modified citrate backbone synthesized by enzymes encoded on the *rhbABCDEF* operon (Lynch *et al*., 2001). After scavenging iron, siderophores bind an outer membrane receptor before being pumped back into the cell (Timofeeva *et al*., 2022). In *S. meliloti*, Rhb1021 uptake depends on an outer membrane receptor encoded by *rhtA* and a permease encoded by *rhtX* (Lynch *et al*., 2001; Cuív *et al*., 2004). While mutations of Rhb1021 biosynthesis genes in ExpR‐deficient *S. meliloti* strains abolish surface motility, it is not affected when only RhtA‐mediated siderophore uptake is prevented, suggesting that the Rhb1021 function for surface motility lies outside the cell (Nogales *et al*., 2010).

Motility is also critical for rhizobia's symbiotic interaction with their legume hosts. Flagella motility helps rhizobia in chemotactic movement to host roots (Caetano‐Anollés *et al*., 1988; Catlow *et al*., 1990; Miller *et al*., 2007; Aroney *et al*., 2021; Compton & Scharf, 2021; Navarro‐Gómez *et al*., 2024), colonization, and attachment to root surfaces (Fujishige *et al*., 2006; Zheng *et al*., 2015), as well as increased competitiveness for nodule occupancy (Ames & Bergman, 1981; Mellor *et al*., 1987; Caetano‐Anollés *et al*., 1988; Miller *et al*., 2007; Aroney *et al*., 2021, 2024). Moreover, a transposon insertion sequencing genetic study in *Rhizobium leguminosarum* found that functional flagella genes enhance bacterial survival and growth in later stages of nodule development (Wheatley *et al*., 2020). But, so far, nonmotile flagella mutants did not appear to significantly affect nodulation or nitrogen fixation, at least in alfalfa or clover species (Ames & Bergman, 1981; Mellor *et al*., 1987). Still, the role of flagella‐dependent or independent motility in rhizobia transcellular host infection remains unclear. Though Rhb1021 biosynthesis seems to enhance biofilm formation and the efficiency of nitrogen fixation in alfalfa (Gill *et al*., 1991; Amaya‐Gómez *et al*., 2015), its impact during rhizobia plant host interaction remains largely unexplored.

In this study, we explored confocal time‐lapse images of fluorescent *S. meliloti* infection events to infer speed and modes of motility used by rhizobia in transcellular IT compartments. To challenge this model, we generated a series of flagella or Rhb1021 biosynthesis and transport mutants in the ExpR‐less *S. meliloti* strain 2011 to tackle the question of how flagella‐dependent and independent paths impact early stages of nodule development and rhizobia colonization in *M. truncatula*. These strains, carrying constitutive β‐galactosidase (*lacZ*) or fluorescent reporters, enabled the quantification of their ability to colonize root hairs or emerging nodules. Altogether, our findings provide novel insights into motility modes privileged by rhizobia to colonize their host roots.

## Materials and Methods

### Mathematical model of infection thread growth

The cell proliferation and motion within the infection thread was modeled using agent‐based modelling mimicking autonomous individual cells with heterogeneous behavior, in a spatially defined environment, that is the Infection Thread, and changing over time. The model is described in detail in Supporting Information Fig. S1 and in brief, in the following. Each cell has its own size represented by an ellipse described by a variable length in μm and a fixed width of 0.4 μm, and can divide when it reaches a maximal length at a certain growth rate in h^−1^. The motion of the cell depends on (1) the migration direction, that is the angle in a 2D plane toward which the bacteria move through the infection thread, and (2) the speed in μm h^−1^ of the cell motion. To simulate the influence of the environment on the cell behavior, a 2D grid was created. Each grid cell corresponds to the local microenvironments encountered by bacteria and may have two properties: (1) the resistance of the environment against bacterial motion due for instance to local modification of the plant cell wall properties or variation in viscosity due to the secretion of exopolysaccharides and (2) the resources available in the local environment and that may be used by the bacteria to pay the cost of proliferation and motility. The model proceeds by calculating for each 10 min time point the following four steps iteratively: step 1,the motion of the cells based on their individual velocity; step 2, the growth of the cell based on their individual growth rate; step 3, the division of the cell if their size and resources allow it; step 4, resolving overlap between cells to push overlapping cells forward. The model was used to test various hypotheses by comparing predictions with experimental measurements.

### Plant materials, bacterial strains, and culture conditions

*Medicago truncatula* Gaertn. Jemalong J5 A17 and the *super numeric nodules*‐2 *sunn‐2* mutant, also in *M. truncatula* Jemalong J5 background (Schnabel *et al*., 2005) were used in this work. The bacterial strains and plasmids used in this study are listed in Table S1. *Escherichia coli* strains were grown at 37°C in lysogeny broth (LB) medium (10 g l^−1^ tryptone, 5 g l^−1^ yeast extract, 5 g l^−1^ NaCl). *Sinorhizobium meliloti* 2011 strains were grown at 28–30°C in tryptone yeast (TY) medium (5 g l^−1^ tryptone, 3 g l^−1^ yeast extract, 0.4 g l^−1^ CaCl_2_). When appropriate, the following antibiotics were added: kanamycin (25 mg l^−1^ for *E. coli*, 100 mg l^−1^ for *S. meliloti*), spectinomycin (50 mg l^−1^ for *E. coli*, 100 mg l^−1^ for *S. meliloti*), streptomycin (200 or 600 mg l^−1^), tetracycline (10 mg l^−1^).

### Generation of *S. meliloti* mutant strains

Synthetic DNA and primers used for the genetic manipulations are listed in Table S2. The plasmid constructs used in this work were generated using standard genetic techniques described in detail in Table S3. Plasmids were transferred from *E. coli* to *S. meliloti* by conjugation. S17‐1 was used for the conjugative transfer of the pXLGD4 and pK18mobsacB‐based plasmids. To introduce the pAB14 plasmid, triparental mating using *E. coli* DH5α and helper strain XL1‐Blue pRK2013 was performed according to the protocol described in Döhlemann *et al*. (2016). Deletions of *fliF–fliR*, *rhbE*, and *rhtA* were generated using the pK18mobsacB suicide plasmid vector, which is nonreplicative in *S. meliloti* and confers both sucrose sensitivity and kanamycin resistance (Schäfer *et al*., 1994). For each deletion, DNA fragments upstream and downstream of the deletion target were inserted into pK18mobsacB (see Tables S2, S3). Double recombinant deletion strains were selected on LB agar supplemented with 10% sucrose, as previously described (Schäfer *et al*., 1994). The plasmid pAB14 was generated by inserting the mScarlet coding sequence and constitutive promoter DNA fragment (oligomers 4 and 5; Table S2) into shuttle vector plasmid pABC–Psyn. The promoter DNA fragment is composed of the promoter region of *SMc06412* (positions −50 to −1 relative to the transcriptional start site), a 5′ untranslated region of *mucR* (positions −271 to −251 relative to the translational start site), and a fragment containing the ribosomal binding site and ATG of *mucR* (positions −20 to +3 relative to the translational start site). Constructs were confirmed by PCR amplification and DNA sequencing.

### Genomic DNA isolation and sequencing

For genomic DNA (gDNA) isolation, *S. meliloti* cell cultures were grown overnight in TY with appropriate antibiotics until they reached an exponential phase, with an OD_600_ between 0.4 and 1. *S. meliloti* gDNA from (*Sm*2011‐lacZ) wild‐type (WT) and *fliF–fliRdel* mutant strains was extracted using a Blood and Cell Culture DNA Kit (Qiagen) with a modified protocol from Mayjonade *et al*. (2016). gDNA from *rhbE* and *rhtA* mutants was purified using the NucleoSpin Microbial DNA Mini Kit (Macherey–Nagel, Düren, Nordrhein‐Westfalen, Germany). Finally, gDNA purity was verified by measuring gDNA concentration and spectrophotometric ratios (*A*_260_ : *A*_280_; *A*_260_ : *A*_230_). *Sm*2011‐lacZ (WT) and *fliF–fliRdel* gDNA at 33 ng μl^−1^ was then used for long‐read sequencing on the Oxford Nanopore Technologies (ONT, Oxford, Oxfordshire, UK) PromethION (Table S4). The *rhbE* and *rhtA* mutant gDNA at 50 ng μl^−1^ was sequenced by Plasmidsaurus using ONT (Table S4). Read mapping to the *Sm*2011 GMI11495 reference genome (Sallet *et al*., 2013) and comparisons for detection of sequence variants were carried out using CLC Genomic Workbench 24.0.1 (Tools: Map Long Reads to Reference 1.2, Basic Variant Detector 2.6 – applied thresholds: Coverage ≥ 10, Count ≥ 6, Frequency ≥ 75%).

### *S. meliloti in vitro* motility assays

Swimming motility assays were performed using cell cultures grown overnight in TY media supplemented with appropriate antibiotics. One milliliter of culture was centrifuged for 2 min at 3000 ***g*** and resuspended in TY. Two microliters of the cell culture was spot inoculated on soft TY 0.3–0.4% agar plates (OD_600_ = 1). Plates were imaged after incubating for 3–4 d at 28°C. Surface motility tests were performed on MM 0.6% agar plates using a modified method adapted from Bernabéu‐Roda *et al*. (2021). 1.2% agar (Pronadisa or Noble Agar; Difco, Becton, Dickinson and Company (BD), Franklin Lakes, NJ, USA) and 2× MM (20 g l^−1^ mannitol, 1.2 g l^−1^ sodium glutamate, 0.6 g l^−1^ KH_2_PO_4_, 0.6 g l^−1^ K_2_HPO_4_, 0.3 g l^−1^ MgSO_4_, 0.1 g l^−1^ NaCl, pH 7.0) were autoclaved separately. After cooling to 60°C, equal volumes of agar and 2× MM solutions were mixed together before adding 340 μl l^−1^ of CaCl_2_ (1 M), 600 μl l^−1^ of FeCl_3_ (10 mg ml^−1^), 200 μl l^−1^ of Biotin (1 mg ml^−1^) and 1 ml l^−1^ of oligoelements (3 mg ml^−1^ H_3_BO_3_, 2.23 mg ml^−1^ MnSO_4_ × H_2_O, 0.287 mg ml^−1^ ZnSO_4_ × 7H_2_O, 0.125 mg ml^−1^ CuSO_4_ × 5H_2_O, 0.065 mg ml^−1^ CoCl_2_ × 6H_2_O, 0.12 mg ml^−1^ NaMoO_4_ × 2H_2_O). After stirring for 3 min, the media was poured on plates (35 ml per plate) and allowed to solidify with closed lids for 1 h. *Sinorhizobium meliloti* strains were grown overnight in TY, diluted the next day to an OD_600_ of 0.2, and grown until they reached an OD_600_ of 1–1.2. Cells from 1 ml of culture were collected by centrifugation for 2 min at 3000 ***g***, washed with 500 μl of 2× MM, collected by centrifugation again, and resuspended in 100 μl of 2× MM. Two microliters of the cell suspensions was spotted in triplicate onto six replicate MM plates and dried with an open lid for 5 min. Plates were incubated at 28–30°C for 3 d and imaged. The surface area (cm^2^) of each spot was measured using ImageJ software.

### Plant growth and bacterial inoculation

After pod dehulling, seeds were scarified by 95% sulfuric acid treatment for 10 min. After washing, bleach treatment (12% sodium hypochlorite) was performed for 2 min to facilitate seed sterilization. After rinsing several times, the seeds were kept in water for a few minutes before being sown on soft agar medium. The Petri dishes sealed with Parafilm were inverted for 2–6 d in the dark at 4°C to synchronize germination. Plates were then incubated at 20°C or 16°C for 17–24 h to induce germination until radicles were > 1 cm long, and then used for IT observation and nodulation experiments.

For IT observation using *in vivo* imaging, germinated seedlings were placed on Fahraeus media plates (12 × 12 cm) supplemented with 0.5 mM NH_4_NO_3_, after their root tips were removed to promote new root emergence. Plates were placed vertically in plastic boxes with black plastic bags covering the roots and kept under controlled 16 h : 8 h, neon light : dark photoperiod conditions and a light intensity of 70 mE s^−1^ m^−2^ at 20°C. After 3 d to 1 wk, plants with new root systems were transferred to nitrogen‐free 0.5% (w/v) phytagel Fahraeus plates supplemented with 50 nM 2‐aminoethoxyvinyl glycine (AVG). Root systems were covered with sterile LUMOX film (Sarstedt, Nümbrecht, Nordrhein‐Westfalen, Germany), and the plates were placed at an inverted tilt so that the roots would grow alongside the film (Fournier *et al*., 2015). After 3 d of nitrogen starvation, plant roots were inoculated with a cell suspension of *Sm*2011‐GFP in experiments used for modeling rhizobia movement or the *Sm*2011‐mScarlet WT or *rhbE* at an OD_600_ of 0.001 for live infection observations. After 30‐min incubation, plants were placed back in the growth chamber and kept in the dark at 20°C or 25°C until microscopic observations. During the observation period, plants were placed in a phytotron under a controlled photoperiod of 16 h : 8 h, neon light : dark at 25°C and 35% humidity.

For nodulation experiments, germinated seedlings of *M. truncatula* A17 were transferred to 8 × 8 × 7 cm pots (three plants per pot) filled with an inert attapulgite substrate (Oil Dri US Special; http://www.oildri.com/) (Fig. S2) or with a mixture of fine vermiculite substrate (*c*. 2/3; Agrigaronne; https://www.agri‐garonne.fr/) and sand (*c*. 1/3; silica 0.7/1.3 mm; Puel) (in experiments shown in main figures). Pots were placed in small glasshouses at 25°C, with a 16‐h photoperiod and a light intensity of 100 mE s^−1^ m^−2^ (for attapulgite experiments) or covered with cellophane and placed in a growth chamber at 25°C : 22°C, day : night, with a 16 h : 8 h, light : dark photoperiod, and a light intensity of 320 μmol s^−1^ m^−2^ and 55% humidity (for vermiculite experiments). Pots were supplemented with nitrogen‐free Fahraeus (for attapulgite experiments) or nitrogen‐free Plant Prod (for vermiculite experiments). After 3 d, plants were inoculated with *Sm*2011‐lacZ WT or mutant bacterial suspensions (OD_600_ 0.1, 4 ml per attapulgite pot and OD_600_ 0.05; 20 ml per vermiculite pot). Nodulated root systems were harvested at 5 or 7 dpi.

### β‐Galactosidase enzymatic assays

Nodulated root systems harvested at 5 or 7 dpi were rinsed in Z buffer (10 mM KCl, 1 mM MgCl_2_, and 0.1 M phosphate buffer, pH 7.0) and fixed in a 1.25% (7 dpi) or 2.5% (5 dpi) glutaraldehyde solution for 1 h (under vacuum) as described previously (Cerri *et al*., 2012). To reveal the constitutive β‐galactosidase activity of the *Sm*2011‐lacZ WT and mutant strains, root samples were rinsed twice with Z buffer and stained in Z buffer with 2 mM X‐gal (5‐bromo‐4‐chloro‐3‐indolyl‐β‐d‐galactopyranoside, W5376C; Thermo Fisher Scientific, Guilford, CT, USA) after incubation at 4°C over the weekend or at 28°C overnight. Blue‐stained roots were cleared for 1 min with a 12% sodium hypochlorite solution before scanning and microscopic observations.

### Microscopy methods

**Fig. 1:**
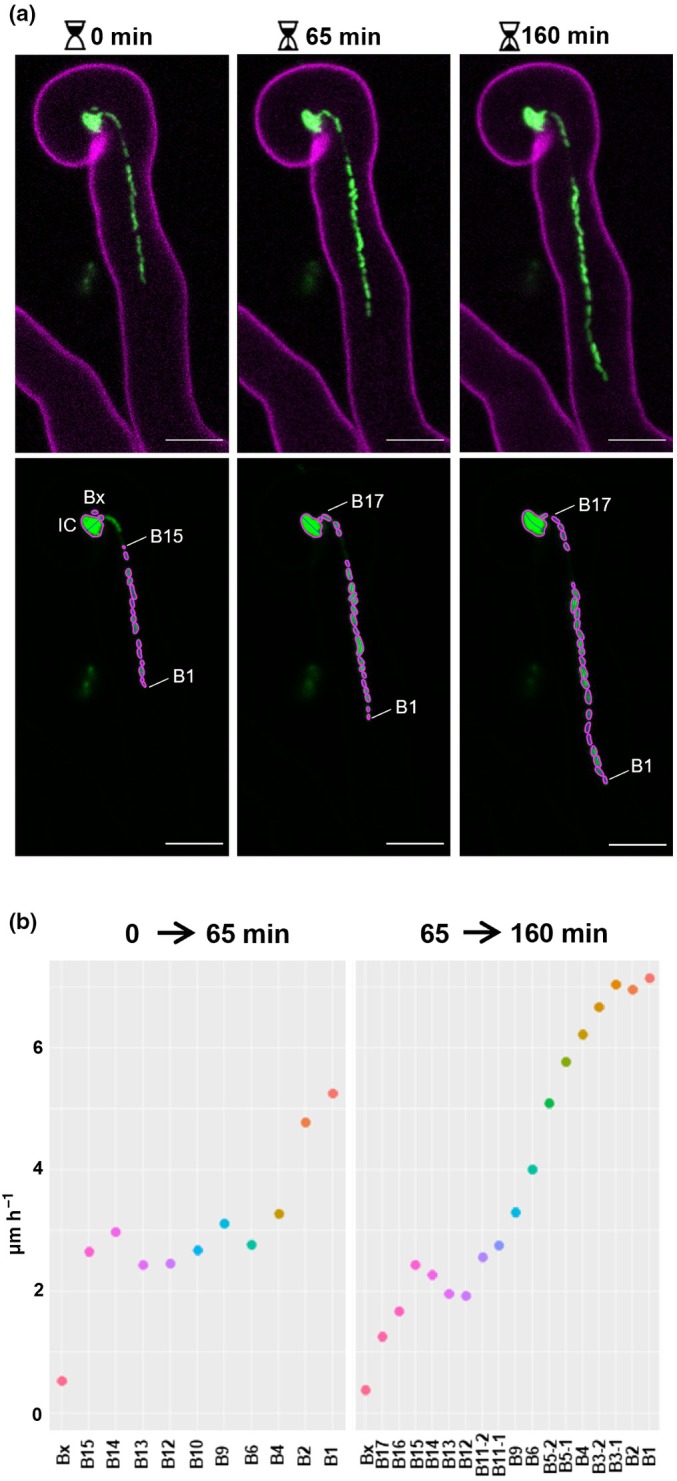
A model of rhizobia motion inside ITs. (a) Tracking *Sinorhizobium meliloti* within ITs. Upper panel: Three successive confocal images of a developing IT within an *Medicago truncatula* root hair were captured at 65‐ and 95‐min intervals. Images of GFP fluorescence from the rhizobia strain (in green) and autofluorescence of the root hair cell wall (in magenta) were merged, and maximal projections of 3 (2 first timepoints) or 6 (last timepoint) confocal sections are shown. Lower panel: The GFP channel of each IT image was used to define regions of interest (ROIs) corresponding to individual rhizobia within the developing IT. Rhizobia were subsequently numbered from the IT tip to the older part, close to the infection chamber (detailed in Supporting Information Fig. S1). The ROIs corresponding to the infection chamber (IC) and a neighboring cell (Bx) were used as a spatial reference. Spatial coordinates of the bacterial cells were determined using the MicrobeJ plugin of ImageJ and used to calculate the speed of each cell over each time interval. Bars, (a) 10 μm. (b) Assessment of the speed of bacterial cells inside ITs. The two graphs present the speeds of some individual rhizobia and their offspring generated during the timeframe of the experiment, between 0 and 65 min, left graph; and between 65 and 160 min, right graph. The speed of each cell was calculated using an agent‐based mathematical model. See also Table S5 and Fig. S1.

For the live analysis of rhizobia within ITs (Fig. 1), *M. truncatula sunn*‐2 plants were grown under LUMOX film on phytagel Fahraeus medium and observed from 2 to 5 dpi with *Sm*2011‐GFP using a Leica TCS SP2 AOBS (Leica Microsystems GmbH, Wetzlar, Hesse, Germany) laser scanning confocal microscope with a ×40 long‐distance water immersion objective (HCX APO L U‐V‐I ×40/0.80 WATER). The argon laser band of 488 nm was used to excite GFP, and a 561 nm diode to excite the cell wall autofluorescence. Specific emission windows used for GFP and autofluorescence signals were 500–530 and 620–720 nm, respectively. Emitted fluorescence was false colored in green (GFP) and magenta (wall autofluorescence). Merged confocal images and maximum projections of several z‐sections were created using the Fiji software. The positions of *S. meliloti* cells within ITs were determined by defining regions of interest (ROIs) and extracting their *x*, *y* coordinates using the MicrobeJ plugin of ImageJ.

For *in vivo* microscopy of *rhbE* and WT Sm2011‐mScarlet infection, *M. truncatula* roots grown under LUMOX film on phytagel Fahraeus media were observed from 2 to 4 dpi with WT or mutant *Sm*2011‐mScarlet strains using a Leica TCS SP8 AOBS laser scanning confocal microscope with a ×40 long‐distance water immersion objective (HCX APO L U‐V‐I ×40/0.80 WATER). A 561 nm diode‐pumped solid‐state (DPSS) laser was used to excite the mScarlet fluorescent protein, and the emitted fluorescence was detected using a hybrid detector (HyD) in the 585–640 nm emission window. Leica Las‐X software was used to record confocal images, and the time‐series images were then analyzed with Fiji software to determine the number of ITs in each root hair. Merged confocal images and maximum projections of several z‐sections were created using the Fiji software for illustrations.

β‐Galactosidase‐stained roots harvested at 7 dpi were scanned with Objectscan 1600 (Microtek, International Inc., Hsinchu, Taiwan). Scanned images were used to analyze nodule numbers, size, and infection levels of the β‐galactosidase‐stained rhizobia, using the Fiji software. Detection of blue β‐galactosidase‐stained nodules was performed with a machine‐learning recognition method with Fiji software (color threshold method). Each nodule or nodule primordium (NP) was defined as a separate region of interest (ROI) and counted for each plant. Specific nodule features, such as area and level of infection (blue intensity of the stained rhizobia β‐galactosidase activity), were then measured for each ROI. A light microscope (AxioPlan II Imaging; Carl Zeiss) was used for capturing images of nodules (Nod) and nodule primordia (NP) at 7 dpi. These categories were distinguished by their appearance and size (Nod are thicker and in a range of 0.100–0.957 mm^2^, while NP are in a range of 0.002–0.099 mm^2^). A nonautomated quantification method was used to count the number of β‐galactosidase‐stained nodules and NPs at 7 dpi in Fig. S2, by using a binocular magnifying glass. Early root hair infection events were quantified in β‐galactosidase‐stained roots harvested at 5 dpi. These were placed on slides, covered with a coverslip, and sealed with nail polish before being scanned with a NanoZoomer 2.ORS (Hamamatsu Photonics France, Massy, Île‐de‐France, France) with a ×20 objective. Fifteen histological sections, spaced 10 μm apart (starting at a depth of 4 μm), were collected per root system and imaged using the Ndp.view2 software. These were used for the illustration or quantification of infection events.

### Graphs and statistical analyses

**Fig. 2:**
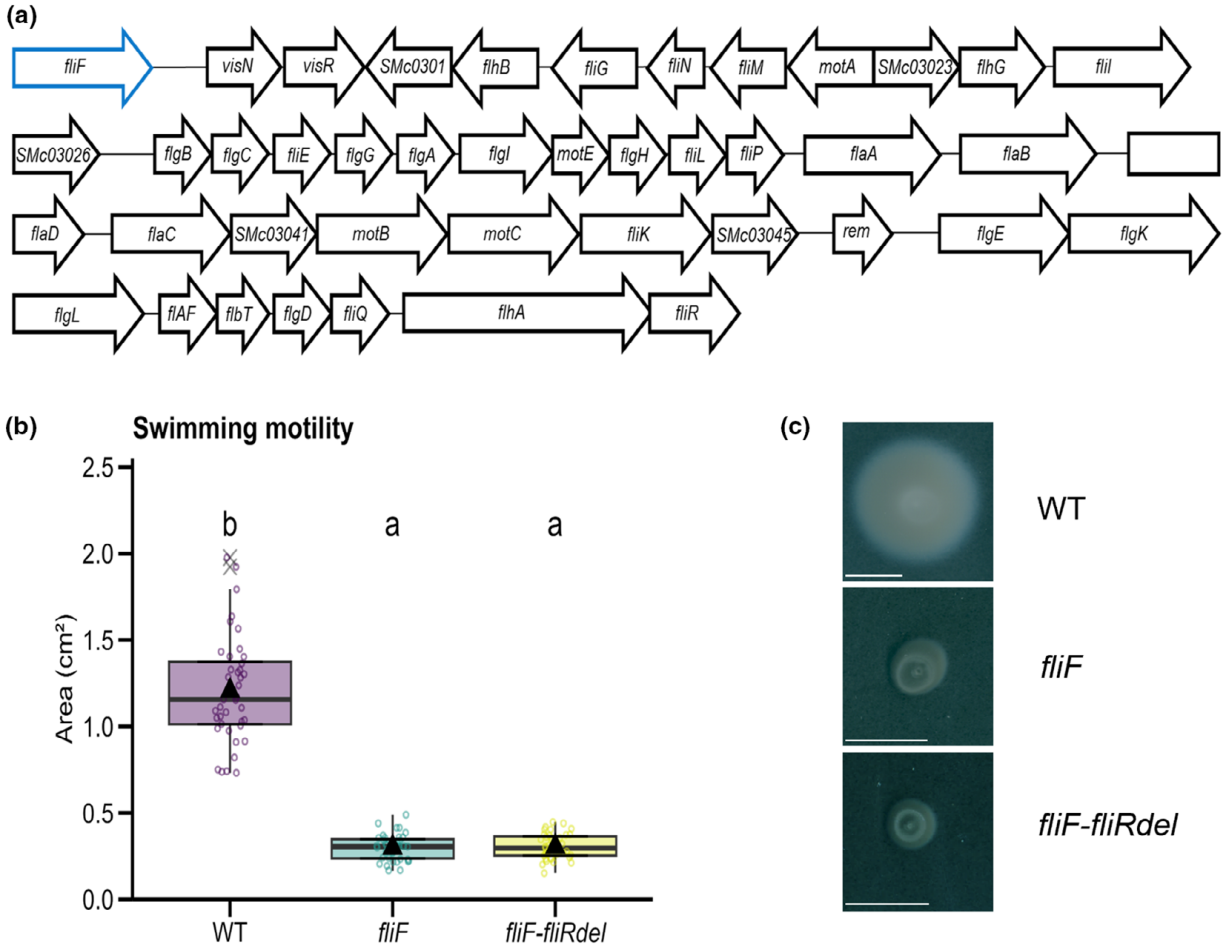
*Sinorhizobium meliloti fliF* and *fliF*–*fliRdel* flagella‐less mutants are impaired in swimming motility *in vitro*. (a) Schematic representation of the *c*. 40 kb region from the *S. meliloti* 2011 (*Sm* 2011) genome, ranging from *fliF* to *fliR*, that was deleted in the *fliF–fliRdel* deletion mutant to abolish flagella biosynthesis and assembly. The *fliF* deletion (in blue) was generated to abolish flagella assembly. (b, c) A swimming motility assay was performed by spot‐inoculation of the *Sm* 2011‐LacZ wild‐type (WT) strain and derived *fliF* and *fliF–fliRdel* deletion mutants on TY (0.3–0.4% agar). Quantification of swimming motility 3–4 dpi (days post inoculation) (WT *n* = 40; *fliF n* = 39; *fliF–fliRdel n* = 40) shown in (b), along with representative images of bacterial motility (c). (b) Box plots show the distribution of values (circles) from three independent experiments (*n* = 39–40 per sample). First and third quartiles (horizontal box edges), minimum and maximum (whisker tips), median (centerline), mean (solid black triangle), and outliers (crosses) are shown. Letters indicate statistically significant differences between groups (*P* < 2.2e‐16, Welch's ANOVA and Games–Howell test). Bars, (c) 1 cm. See also Supporting Information Table S2.

Graphical representation and statistical analyses of the data were performed using R. Data are represented either as box plots or stacked bar charts. The normal distribution of the data was assessed using the Shapiro–Wilk test, and in the case of a nonnormal data distribution, a transformation was performed to normalize the data distribution if possible (Log_10_ or BoxCox). In the case of normal distribution, homogeneity of variance was evaluated using Fisher or Bartlett tests. For normal data distribution, data were analyzed with parametric statistical tests (*t*‐test, one‐way ANOVA, Tukey test, Welsh's ANOVA, and Games–Howell test), whereas nonparametric tests (Mann–Whitney, Kruskal–Wallis, and Dunn test) were used for data with a nonnormal distribution. Fisher's tests were used to analyze data in contingency tables. Figure legends indicate the number of replicates and significance *P*‐value levels, as well as the total number of individuals analyzed (*n*). In detail for each figure, the data were analyzed as follows: Swimming motility assay values for WT and flagella‐less mutants in Fig. 2 follow a normal distribution and the variances were not homogeneous, so a Welch's ANOVA followed by a Games–Howell test was performed (*F* = 164.17, *P* < 2.2e‐16).

**Fig. 3:**
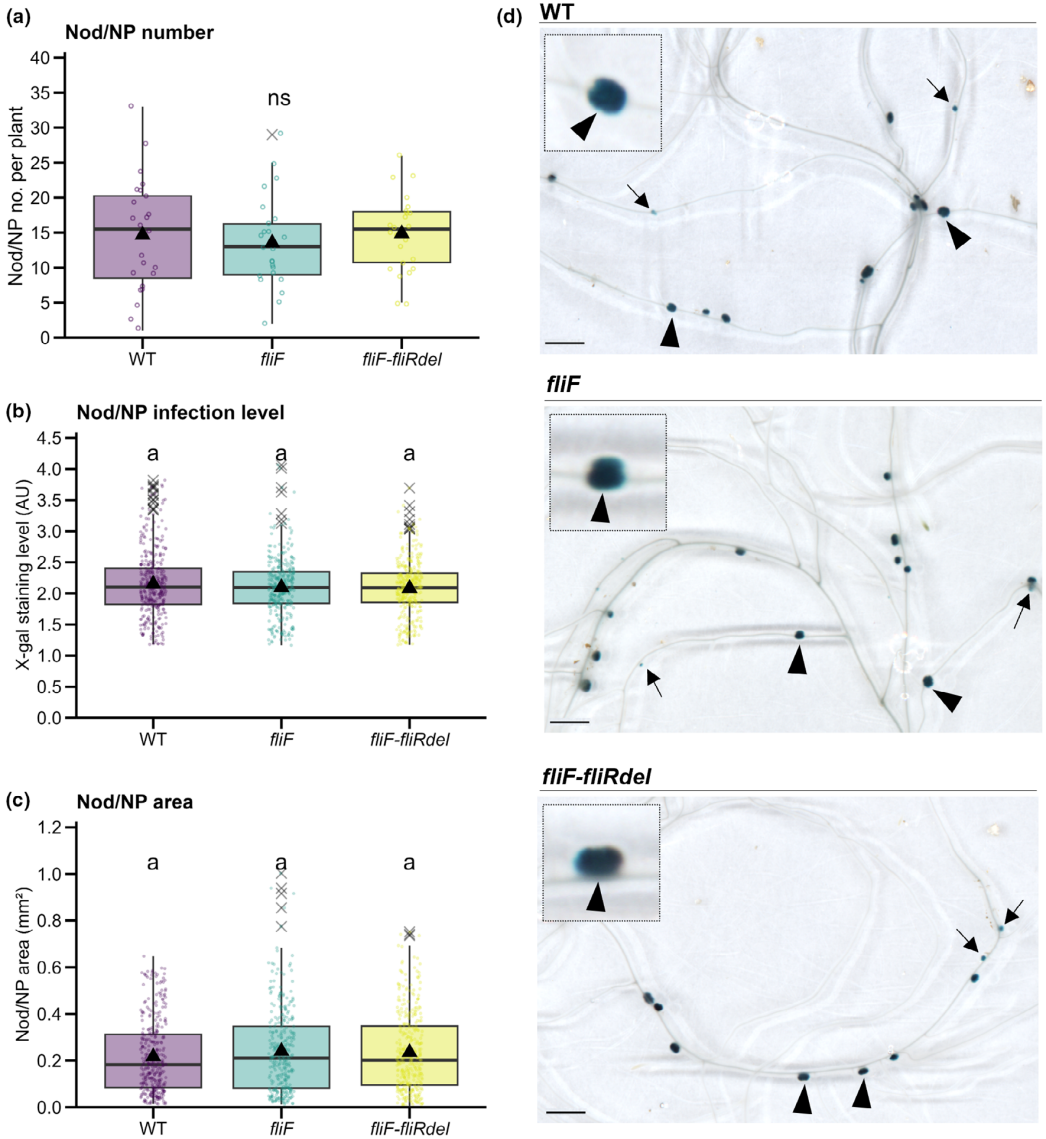
*Sinorhizobium meliloti fliF* and *fliF–fliRdel* flagella motility mutants form nodules that are fully infected in *Medicago truncatula*. The impact of flagella mutants in early nodule development and infection was quantified in X‐gal‐stained *M. truncatula* root systems inoculated with *lacZ*‐expressing *S. meliloti* WT or *fliF* and *fliF–fliRdel* mutant strains at 7 dpi (days post inoculation). (a–c) Number of nodules (Nod) and nodule primordia (NP) per plant (a), infection level (X‐gal staining intensity) per Nod/NP (b), and Nod/NP area (c) were quantified at 7 dpi in scanned images of nodulated plants’ root systems (WT *n* = 24; *fliF n* = 24; *fliF–fliRdel n* = 24 in (a) or individual Nod/NP (WT *n* = 352; *fliF n* = 325; *fliF–fliRdel n* = 357 in (b, c)) from two independent experiments. (d) Representative images of scanned nodulated root systems. Close‐up views of X‐gal‐stained nodules (in blue) are shown in the top left squares. Nodules (arrowheads) and NP (arrows) are indicated. Box plots in (a–c) show the distribution of values (circles), first and third quartiles (horizontal box edges), minimum and maximum (whisker tips), median (centerline), mean (solid black triangle), and outliers (crosses). ns (nonsignificant) (a) indicate no statistical difference relative to WT (*P* = 0.766, one‐way ANOVA). Classes with the same letter (b, c) are not significantly different (*P* = 0.6427096 in b, *P* = 0.3148374 in c, Kruskal–Wallis α = 5%). Bars, (d) 3 mm. See also Supporting Information Fig. S2.

**Fig. 4:**
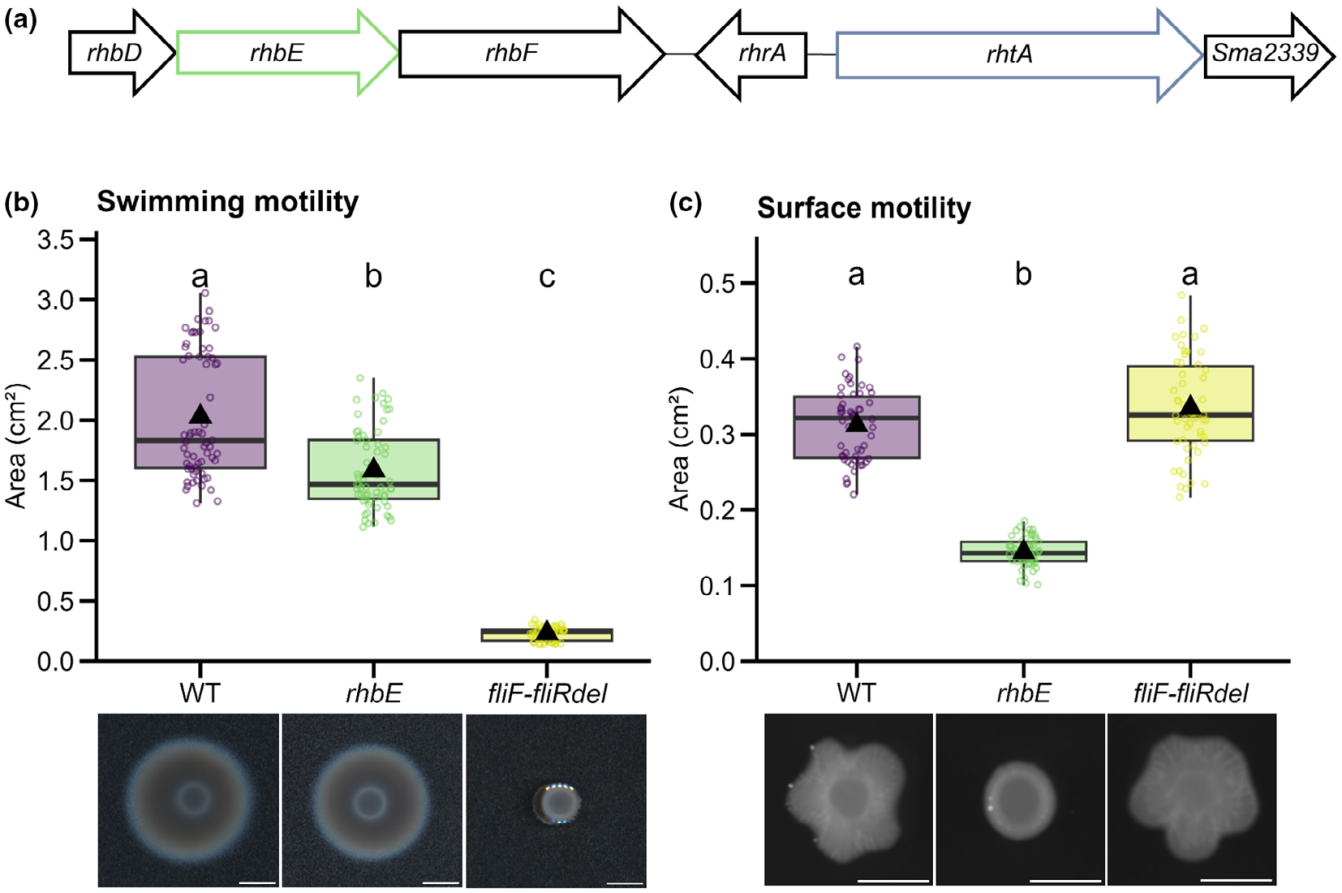
The *Sinorhizobium meliloti* siderophore rhizobactin 1021 biosynthetic gene *rhbE* mutant is impaired in surface motility *in vitro*. (a) Schematic representation of the genomic region of *S. meliloti 2011* comprising *rhbE* and *rhtA* genes, which were deleted in the *S. meliloti 2011*‐lac Z strain to abolish rhizobactin 1021 biosynthesis or its utilization, respectively. Deleted genes are represented by green and blue boxes. (b, c) Swimming motility (b) and surface motility (c) assays were performed by spot‐inoculation of *S. meliloti* WT, *rhbE*, and *fliF–fliRdel* strains on plates with TY 0.3% agar (b) or MM 0.6% agar (c). Box blots show the distribution of values (circles) obtained after ImageJ quantification of bacterial colony growth area 3–4 d (b) or 3 d (c) after spot inoculation from 16 to 24 samples (b, c) technical replicates from three independent experiments (WT *n* = 64; *rhbE n* = 62; *fliF–fliRdel n* = 56 in b, WT *n* = 54; *rhbE n* = 54; *fliF–fliRdel n* = 54 in c). First and third quartiles (horizontal box edges), minimum and maximum (whisker tips), median (centerline), mean (solid black triangle), and outliers (crosses) are shown in box plots. Letters indicate statistically significant differences between groups (*P* < 2.2e‐16 in b, Kruskal–Wallis α = 5%; *P* < 2.2e‐16, Welch's ANOVA, and Games–Howell test in c). Representative images of bacterial growth areas on plates are shown in the graphs below. Bars, (b, c) 4 mm. See also Supporting Information Table S3.

**Fig. 5:**
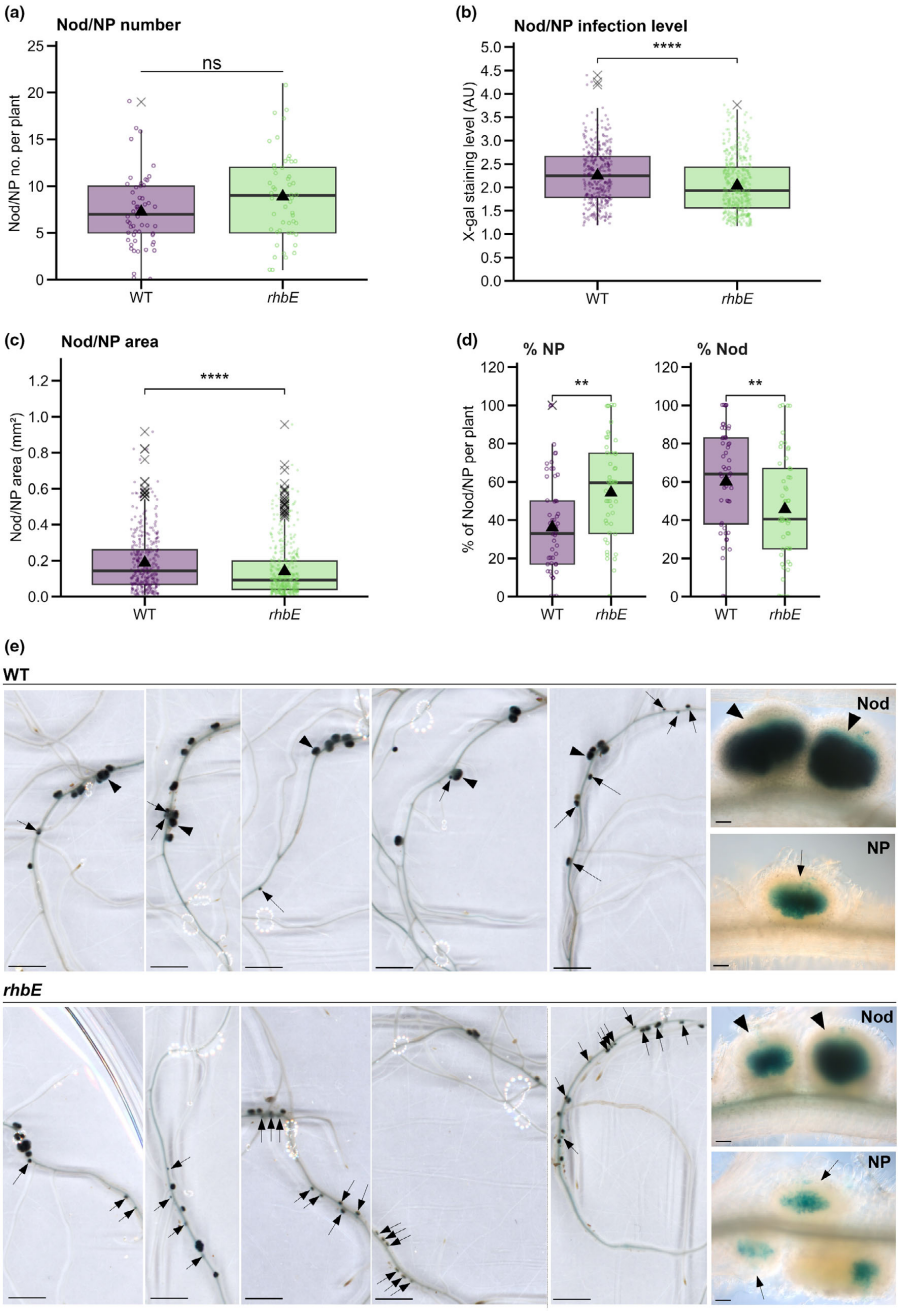
A mutation in the *rhbE* rhizobactin siderophore 1021 biosynthetic gene affects early rhizobia‐induced nodule development and colonization. The impact of *rhbE* mutation in early nodule development and infection was quantified in X‐gal‐stained *Medicago truncatula* root systems inoculated with *lacZ*‐expressing *Sinorhizobium meliloti* WT or *rhbE* mutant strains (visualized in blue) at 7 dpi (days post inoculation). (a–c) Number of nodules (Nod) and nodule primordia (NP) (a), Nod/NP infection level (X‐gal staining intensity) (b) and Nod/NP area (mm^2^) (c) were quantified in individual root systems (WT *n* = 53; *rhbE n* = 52 in a) or individual Nod/NP (WT *n* = 384; *rhbE n* = 462, in b, c). (d) Percentage of NP (area < 0.100 mm^2^) and Nod (area ≥ 0.100 mm^2^) were quantified per plant (WT *n* = 53; *rhbE n* = 52). Box plots (a–d) show the distribution of values (circles) from three independent experiments. First and third quartiles (horizontal box edges), minimum and maximum (whisker tips), median (centerline), mean (solid black triangle), and outliers (crosses) are shown. ns (nonsignificant) (a) indicate no statistical difference relative to WT (*P* = 0.05708, two‐tailed Student's *t*‐test). Asterisks (b–d) point to statistical differences in *rhbE* relative to WT samples (*P* = 6.389e‐08 in b, *P* = 1.599e‐08 in c, *P* = 0.001205 for % NP and *P* = 0.00812 for % Nod in d, Mann–Whitney test). (e) Representative images of *M. truncatula* root systems nodulated with WT or *rhbE* strains from experiments in a–c. Nod (arrowheads) and NP (arrows) are indicated, and their close‐up views are shown in the right corner (a–d). Bars: (e) 3 mm; 100 μm (close‐up images). See also Supporting Information Fig. S2.

**Fig. 6:**
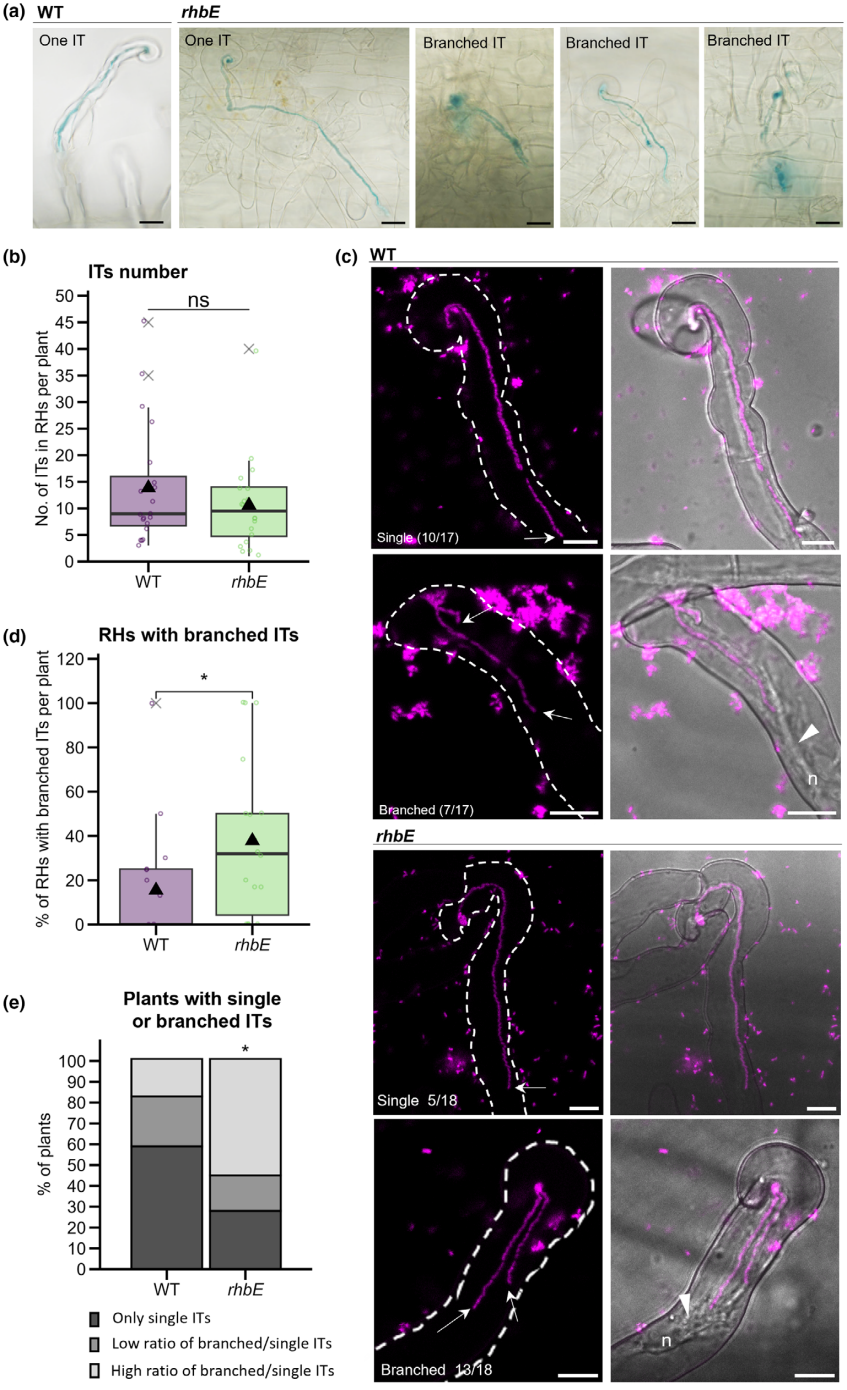
Impact of *rhbE* mutation on IT root hair development in *Medicago truncatula*. The impact of *rhbE* mutation in early development of ITs in root hairs was analysed in *M. truncatula* X‐gal‐stained root systems (a, b) after inoculation with *lacZ*‐expressing *Sinorhizobium meliloti* WT or *rhbE* mutant strains (visualized in blue) or *in vivo* (c–e) in roots inoculated with *S. meliloti* WT or *rhbE* mutant strains expressing a mScarlet fluorescent reporter. (a, b) Number of ITs in root hairs was quantified by counting root hair lacZ‐stained rhizobia infection events (illustrated in a) WT and mutant samples on bright field microscopy images taken from slide‐scanned rhizobia‐inoculated root systems at 5 dpi (days post inoculation) from two independent experiments (WT *n* = 20; *rhbE n* = 20) (b). (c–e) Confocal microscopy analysis of early IT development using mScarlet *S. meliloti* WT or *rhbE* mutant strains was carried out *in vivo* from 2 to 4 dpi. (c) Representative confocal fluorescent and merge images of RHs with single or branched ITs (visualized in magenta). Arrows indicate IT tips, which are linked to the nucleus (n) by a cytoplasmic bridge (white arrowhead). Proportion of RHs with branched ITs per plant (d) or of individual plants having only single ITs or having a different ratio of branched vs single ITs (e): low (≤ 1 : 4 branched vs single ITs) or high (≥ 1 : 3 to 1 : 0 branched vs single ITs). Data in (d, e) are from four independent experiments (WT *n* = 17; *rhbE n* = 18). Box plots in (b, d, e) show the distribution of values (circles) from 2 (b) or 3 (c) independent experiments. First and third quartiles (horizontal box edges), minimum and maximum (whisker tips), median (centerline), mean (solid black triangle), and outliers (crosses) are shown. ns (nonsignificant) (b) indicate no statistical difference relative to WT (*P* = 0.2525, two‐tailed Student's *t*‐test). Asterisks (d, e) point to statistical differences in mutant relative to WT (*P* = 0.03273 in d, Mann–Whitney test; *P* = 0.0354 for 26–100% in e, two‐tailed Fisher's exact tests). Bars: (a) 20 μm; (e) 10 μm.

Values of nodule (Nod) and nodule primordia (NP) number formed by WT and flagella‐less mutants in Fig. 3 follow a normal distribution and variance homogeneity, so a one‐way ANOVA was carried out (*F* = 0.268, *P* = 0.766). Values of Nod/NP infection level and area do not follow a normal distribution and were thus analyzed using a Kruskal–Wallis test (*K* = 0.8841246, *P* = 0.6427096 for infection level values or *K* = 2.311398, *P* = 0.3148374 for area values). Values for Nod/NP number and infected Nod/NP number in Fig. S2 follow a normal distribution and variance homogeneity, so a one‐way ANOVA was performed (*F* = 0.113, *P* = 0.894 in a; *F* = 0.225, *P* = 0.799 in b). Swimming motility values for WT, *rhbE*, and *fliF–fliRdel* in Fig. 4 do not follow a normal distribution, so a Kruskal–Wallis test was performed (*K* = 128.0703, *P* < 2.2e‐16). Surface motility values for WT, *rhbE*, and *fliF–fliRdel* in Fig. 4 follow a normal distribution, and the variances were not homogeneous; hence, Welch's ANOVA followed by a Games–Howell test was performed (*F* = 446.14, *P* < 2.2e‐16). BoxCox‐transformed, Nod/NP number values for WT, *rhbE* (λ = 0.5858586), in Fig. 5, show normal distribution and variance homogeneity and thus were analyzed using a two‐tailed Student's *t*‐test (*T* = 1.9243, *P* = 0.05708). Values for Nod/NP infection level and area as well as percentage of NP or Nod for WT and *rhbE* in Fig. 5 do not follow a normal distribution, so Mann–Whitney tests were applied (*W* = 107 840, *P* = 6.389e‐08 for Nod/NP infection level; *W* = 108 700, *P* = 1.599e‐08 for Nod/NP area; *W* = 873, *P* = 0.001205 for % NP and *W* = 1791, *P* = 0.00812 for % Nod). Values in Fig. S3 do not follow a normal distribution; hence, a Kruskal–Wallis test was carried out (*K* = 23.95754, *P* = 6.276046e‐06). Box–Cox‐transformed values (λ = 0.02020202) of IT number for WT and *rhbE* in Fig. 6 show normal distribution and variance homogeneity; hence, a two‐tailed Student's *t*‐test (*T* = −1.1619, *P* = 0.2525). In Fig. 6, values of RHs with branched ITs for WT and *rhbE* do not follow a normal distribution and were thus analyzed using a Mann–Whitney test (*W* = 90.5, *P* = 0.03273) complemented by a permutation test (10 000 permutations) (*P* = 0.0461) to confirm the robustness of the statistical inference. A graph was created from a contingency table reporting the relative proportion of individual plants having only single ITs or having a different ratio of branched vs single ITs: low (≤ 1 : 4 branched vs single ITs) or high (≥ 1 : 3 to 1 : 0 branched vs single ITs). Fisher's exact tests allowed us to compare the relative proportions of these categories between the WT and *rhbE* mutant strain (*P* = 0.0922 for single ITs; *P* = 0.691 for low ratio; *P* = 0.0354 for high ratio).

## Results

### IT infection kinetics and modelling suggest slow‐motion motility of *S. meliloti* cells in elongating ITs

Previous microscopy studies suggested that elongation of the rhizobia bacterial file within ITs likely involves slow movement modes (Gage, 2002; Fournier *et al*., 2008). To quantitatively assess the motility speed used by rhizobia inside ITs and to test possible associated motility mechanisms, we built a multi‐agent mathematical model mimicking the motion and proliferation of rhizobia bacterial cells. Hence, the contribution of different motility mechanisms like swimming or swarming (active) can be decoupled from sliding or surfing (passive, see Table S5). To this end, we used a series of 3–5 successive images, acquired at intervals of 40–100 min, of individual developing ITs (*n* = 5). Thanks to the localization of individual bacterial cells over time (see Figs 1, S1), we could calculate the distances traveled by each bacterium over time and formulate modelling hypotheses (Fig. 1). The model was parametrized with the shape of the IT and position of the bacterial cells using bright field and confocal microscopy images and fit to experimental data to infer contribution of various types of motilities. Analysis of the experimental data, thanks to the model, revealed that the cells at the forefront of the IT are more mobile than those further back, in accordance with Gage (2004). An average speed of 3.8 ± 1.7 μm h^−1^ was found for the forefront six cells in each experiment, and the cells dividing harbor a doubling time with a minimal value of 3.6 h. Thus, a maximal motion of 0.48 μm h^−1^ can be due to cell division alone, which could contribute to sliding motility. This level represents *c*. 15% of the speed of the most mobile cells. Thus, sliding‐independent motility may represent, on average *c*. 3.3 μm h^−1^ (85%). The order of magnitude of the speed of the cells was *c*. 4 μm h^−1^, and between 2 and 6 μm h^−1^.

We compared the sliding‐independent motility contribution (*c*. 3.3 μm h^−1^) with previously published speeds of bacterial motility (Table S5). In liquid or semi‐solid media, flagella‐dependent motility (i.e. swimming and swarming) propel bacterial cells at very high speeds (5–10 mm h^−1^) (Table S5) up to three orders of magnitude higher than the speed observed in ITs. Entropy‐driving motility (i.e. surfing) is slower than swimming and swarming (0.5–1 mm h^−1^; Gao *et al*., 2012; Dilanji *et al*., 2014), but still an order of magnitude higher than the speed observed in the IT (Dilanji *et al*., 2014). Considering these discrepancies in speed, we hypothesized that flagella‐independent motility might be involved in the progression of rhizobia within early ITs. However, it is possible that rhizobia use flagella in the IT, but that spatial constraints or the viscosity of the IT lumen limit its speed.

### *S. meliloti fliF* and *fliR–fliRdel* flagella mutants are impaired in swimming motility *in vitro*

To investigate the role of flagella during host infection, the 39 838 bp chromosomal flagellar regulon region, extending from *fliF* (encoding the flagellar MS‐ring rotor protein) to *fliR* (encoding a probable flagellar biosynthesis protein) (Sourjik *et al*., 2000) was deleted in the genome of *S. meliloti* 2011 (*Sm*2011), resulting in the *fliF–fliRdel* markerless mutant strain (Fig. 2a). A second deletion focused on *fliF* (in blue in Fig. 2a), which encodes a key component of the MS ring at the core of the flagella motor's basal body, was also generated. As flagellar structure is initiated by the assembly of the FliF protein, the *fliF* mutation specifically abolishes flagella formation. Genome sequence comparison of the reference *Sm*2011 wild‐type (WT) and the *fliF–fliRdel* strain confirmed the deletion of the flagellar regulon region (Fig. 2a; Table S6).

To later facilitate histochemical visualization of the strains during *in planta* infection, a plasmid constitutively expressing a β‐galactosidase (*lacZ*) reporter (pXLGD4; Leong *et al*., 1985) was introduced in these strains, which are hereafter referred to as *Sm*2011*‐lacZ* WT, *fliF*, or *fliF–fliRdel*. To address their swimming abilities, swimming motility tests were performed on 0.3–0.4% agar TY plates by measuring the colony migration growth zone at 3–4 dpi. The WT strain spread through the medium, covering an area approximately four times larger than that of *fliF* and *fliF–fliRdel* mutants (Fig. 2b,c). These results confirm the swimming ability of the WT strain over time and show that swimming motility is abolished in the *fliF* and *fliF–fliRdel* strains *in vitro*.

In summary, these data demonstrate that both flagella mutants exhibit equivalent impairments in flagella‐dependent swimming motility *in vitro*, regardless of whether the deletion affects a single gene responsible for flagellum assembly (*fliF*) or the entire flagellar regulon (*fliF–fliRdel*).

### *S. meliloti fliF* and *fliF–fliRdel* flagella motility mutants do not exhibit early nodule development and colonization phenotypes *in planta*

While the motility of rhizobia in free‐living conditions has been extensively studied (Gotz & Schmitt, 1987; Nogales *et al*., 2012; Wadhwa & Berg, 2022), the specific form of motility they employ to navigate inside the confined IT environment remains unknown. The motility model outlined in Fig. 1 suggested minimal contribution of flagella‐dependent motility inside ITs. To investigate this, we compared the ability of lacZ‐tagged *fliF* and *fliF–fliRdel* flagella‐less mutants to induce and colonize nodules on *M. truncatula* roots relative to the *Sm*2011*‐lacZ* WT control. By improving a recently developed method (Guillory *et al*., 2024) based on ImageJ quantification of X‐gal‐stained root systems infected with *lacZ*‐expressing *S. meliloti* strains, we were able to monitor the number and size of nodules (Nod) and nodule primordia (NP) as well as their infection level in root systems of individual plants. A similar number of nodules (Nod) and nodule primordia (NP) were formed in plants inoculated with flagella‐less mutants compared with the WT control (Fig. 3a). Moreover, nodules and NP formed with flagella‐less mutants were well infected and displayed no obvious signs of developmental defects compared with the WT control (Fig. 3b–d). Similar conclusions were reached when plants were grown under another growth condition and quantified via nonautomated, user‐based microscopy counting (Fig. S2).

In conclusion, flagella‐less mutants do not exhibit significant differences in nodule formation or infection levels in *M. truncatula* compared with the control strain when flood‐inoculated, regardless of growth conditions or quantification methods. These results are consistent with the model (Fig. 1) and previous studies in other legume species (Ames & Bergman, 1981; Mellor *et al*., 1987).

### Mutation in the *rhbE* rhizobactin siderophore 1021 biosynthetic gene affects *S. meliloti* surface motility *in vitro* and *in planta* root nodule colonization

The model (Fig. 1) and experimental data obtained with flagella‐less mutants (Figs 3, S2) both support that *S. meliloti* uses flagella‐independent motility modes to move inside ITs during infection of their legume host. Regardless of flagella, surface motility in *S. meliloti* relies on the secretion of surfactant‐like compounds, such as EPS II and Rhb1021, to reduce surface friction (Nogales *et al*., 2012; Dilanji *et al*., 2014). As the *S. meliloti* strain 2011 used in this study is impaired in EPS II production (Nogales *et al*., 2012), we focused here on evaluating the role of Rhb1021 in the symbiotic interaction. Mutations in *rhb* genes were previously shown to block the synthesis of Rhb1021 and to render *S. meliloti* 2011 nonmotile on semisolid MM (Lynch *et al*., 2001; Nogales *et al*., 2010, 2012). Here, we created a markerless deletion of *rhbE* (Fig. 4a) using homologous recombination, which was subsequently confirmed by genomic sequencing (Table S7). Unlike the flagella‐less *fliF–fliRdel* mutant, the *rhbE* mutant could still swim, albeit slightly less efficiently than the WT strain, in TY agar 0.3% plates comparable to the WT control at 3–4 dpi (Fig. 4b). Conversely, *rhbE* showed significant impairment of surface translocation on semisolid MM plates compared with WT and *fliF–fliRdel* strains at 3 dpi (Fig. 4c). Together, these results confirm the involvement of Rhb1021 in promoting passive surface motility of *S. meliloti*.

To investigate how impairment in Rhb1021 production could impact root nodulation, we examined the ability of the *rhbE* mutant strain (also expressing *LacZ*) to colonize and develop nodules in *M. truncatula* relative to the WT control, using the same ImageJ quantification method as used with the flagella mutants (Fig. 3). At 7 dpi, the total number of nodules (Nod) and nodule primordia (NP) formed per root system with the *rhbE* strain was comparable to those formed with the WT strain (Fig. 5a). However, infection levels and overall sizes of *rhbE* Nod and NPs (Fig. 5b,c) were consistently reduced compared with those in WT plants. A detailed analysis of Nod and NP distribution per individual plants revealed the overrepresentation of small and under‐infected NPs (arrows) in *rhbE* and the opposite overrepresentation of larger, well‐infected nodules (arrowheads) in WT‐inoculated plants (Fig. 5d,e). Together, these results suggest that Rhb1021 biosynthesis by rhizobia contributes to root nodule colonization.

As secreted siderophores can also function as high‐affinity iron chelators to acquire iron when it is scarce in the environment (Timofeeva *et al*., 2022), it remained to be established if the *in planta* phenotype of the *rhbE* strain was due to a defect in rhizobactin‐mediated sliding motility or rather in iron uptake. To distinguish between these possibilities, we generated a markerless deletion of *rhtA* encoding the Rhb1021 outer membrane receptor (Fig. 4a), which is expected to affect Rhb1021 utilization to scavenge iron but not the ability to synthesize it (Lynch *et al*., 2001). This markerless deletion was subsequently confirmed by genomic sequencing (Table S8). As in previous analyses (Fig. 5c), Nod and NPs in roots inoculated with *rhbE* were significantly less infected (Fig. S3). In contrast to the *rhbE* mutant, nodules and NPs formed in *rhtA*‐strain inoculated roots showed similar infection levels to those formed in WT‐inoculated roots (Fig. S3). Collectively, these findings imply that early defects in nodule development and infection by the *rhbE* Rhb1021 biosynthetic mutant are likely attributable to a defect in sliding motility.

### The *rhbE* mutation favors the development of branched ITs in‐root hairs

To further dissect the observed *rhbE* mutant infection phenotype, we closely inspected early root hair infection events driven by *rhbE* compared with the WT strain. Using rhizobia strains expressing the *lacZ* reporter, ITs formed in *M. truncatula* root hairs 5 dpi were visualized and quantified (Fig. 6a,b). The number of ITs formed per root hair was not significantly different between the two strains (Fig. 6b). Nevertheless, RHs with branched ITs (two or three branches) per root hair were frequently observed in root systems inoculated with the *rhbE* strain (Fig. 6a). To better quantify these early infection events, we used WT and *rhbE* strains constitutively expressing an mScarlet fluorescent reporter for visualizing fluorescent‐labeled ITs in root hairs 2–4 dpi using *in vivo* confocal microscopy (Fig. 6c–e). Growing ITs, recognized by the presence of a cytoplasmic bridge (arrowhead) or the nucleus (n) located in front of the tip (arrow), were imaged in both *rhbE* and WT‐inoculated plants (Fig. 6c) for subsequent ImageJ‐based quantification. Roots inoculated with the *rhbE* strain showed preferential formation of branched ITs per RH compared with the WT control (Fig. 6d). Furthermore, a higher proportion of branched ITs was found in individual *rhbE* plants than in the WT control (Fig. 6c,d). Indeed, most WT plants had only single ITs (60%, 10/17), whereas this category represented only 30% of *rhbE* plants (5/18). Similar proportions of plants (24% in WT, 4/17 and 17% in *rhbE*, 3/18) exhibited low ratios of branched ITs (≤ 25 % branched ITs). By contrast, plants with a high proportion of branched ITs (> 25 % branched ITs) were overrepresented for the *rhbE* strain (10/18) compared with WT (3/17). Together, these results show that the *rhbE* mutation partially impacts the development of ITs in root hairs, which may explain the observed reduced host colonization. Mutation of *rhbE* strikingly promotes IT branching in RHs, raising the question of the need for Rhb1021 for guiding tip directional growth of ITs.

## Discussion

In this study, we integrated mathematical modeling, live cell imaging, and *in planta* phenotypic analyses of bacterial mutants with reduced motility to elucidate motility modes used by rhizobia during early stages of colonization of their legume host. Focusing on the *S. meliloti*–*M. truncatula* symbiotic model system, our findings indicate that flagella‐less *S. meliloti fliF* and *fliF–fliRdel* mutants, which are abolished in swimming motility *in vitro*, are not impaired in nodule initiation, development, or infection (Figs 2, 3). By contrast, an *S. meliloti rhbE* mutant, which is blocked in Rhb1021 biosynthesis and affected in surface motility (Nogales *et al*., 2010, 2012 and this work; Figs 4, 5, 6), can initiate nodule primordia and nodule formation, but these are affected in their development and infection level. These findings are consistent with the estimated model (Fig. 1) that rhizobia preferentially rely on passive flagella‐independent paths to colonize their host and provide new evidence that siderophore Rhb1021 biosynthesis by rhizobia plays a role in legume host infection.

Here, we provided a detailed phenotypic characterization and quantification of early infection and nodule development phenotypes of flagella‐less mutants *fliF* and *fliF–fliRdel*, not done before in rhizobia–legume symbiosis. The lack of an obvious symbiotic phenotype of flagella‐less mutants *fliF* and *fliF–fliRdel* in *M. truncatula* when bacteria are flood inoculated (Fig. 3) is in line with previous studies in other rhizobia–legume symbiotic interactions showing that flagella are not essential for late nodule development (Ames & Bergman, 1981; Mellor *et al*., 1987; Salas *et al*., 2017; Navarro‐Gómez *et al*., 2024). While motile strains are more competitive for nodule occupancy compared with nonmotile strains (reviewed in Aroney *et al*., 2021), this advantage likely comes at earlier stages, such as movement toward the root (Mellor *et al*., 1987; Bernabéu‐Roda *et al*., 2015; Navarro‐Gómez *et al*., 2024), attachment (Fujishige *et al*., 2006; Zheng *et al*., 2015), and spreading on the root surface (Caetano‐Anollés *et al*., 1988). Nevertheless, a transposon insertion sequencing genetic study showed that flagella‐less mutants are impacted in their survival and growth at later stages in nodules (Wheatley *et al*., 2020). Although our results do not support a prominent role of flagella during early host colonization, we cannot completely rule out a potential synergistic role in facilitating early root infection. Analyzing the infection phenotypes of double *rhbE* and flagella‐less mutants could help clarify this. Further high‐resolution *in vivo* biotracking tools (Ozer *et al*., 2021) could provide complementary relevant information on when flagella are actually lost during the symbiotic colonization of the host.

Our experimental data and model (Figs 1, 3) suggest that the collective movement of rhizobia through the IT compartment can occur independently of flagella. Our model favors slow (2–6 μm h^−1^) over rapid (5 mm h^−1^) (Table S5) movement of rhizobia within early root hair IT compartments. This corresponds more closely to speeds associated with passive forms of motility that are not driven by the propulsive force generated by flagella (i.e. sliding and surfing). This is consistent with the observation that at high cell population densities, as inside ITs, genes associated with the flagellar regulon are repressed (Gurich & González, 2009). Building on previous observations that *S. meliloti* likely do not use twitching or gliding (Zatakia *et al*., 2014; Wadhwa & Berg, 2022) but synthesize the siderophore Rhb1021 for passive surface motility (Nogales *et al*., 2010, 2012; Bernabéu‐Roda *et al*., 2015), we conducted a genetic study of the role of Rhb1021 biosynthesis and uptake in the *S. meliloti*–*Medicago* symbiosis. Phenotypic characterization of the Rhb1021 biosynthetic *S. meliloti rhbE* mutant provided novel evidence for a role of Rhb1021 in proper development and colonization of NP and nodules in *Medicago*. As infection and nodule development are interconnected processes (Xiao *et al*., 2014), we believe that the nodule size developmental defects observed in *rhbE*‐inoculated plants might be a consequence of the impaired NP and nodule colonization (Fig. 5). Defective nodule phenotypes of the *rhbE* strain could possibly impact later stages of nodule differentiation and functioning, which could explain the reduced nitrogen fixation efficiency previously described for another Rhb1021 biosynthesis mutant (Gill *et al*., 1991). As no obvious nodulation phenotypes were observed with the *rhtA* mutant (Fig. S3), which can synthesize Rhb1021 but cannot use it to acquire iron, we can confidently conclude that the symbiotic phenotypes observed with *rhbE* are not due to an iron‐scavenging issue but rather due to the absence of Rhb1021 *per se*, which likely permits optimal motility during host infection. These results are consistent with a previous study showing that a *R. leguminosarum* mutant impaired in siderophore uptake but not biosynthesis was unaffected in its ability to induce N_2_‐fixing nodules in pea (Stevens *et al*., 1999).

In line with previous studies (Nogales *et al*., 2010, 2012), the Rhb1021 biosynthesis *rhbE* mutant produced in this study showed impaired surface motility in semisolid MM medium (Fig. 4). As for other surfactants (Burch *et al*., 2012), it has been proposed that Rhb1021 production could be coordinated with flagellar assembly and thus be affected in flagella‐less mutants (Bernabéu‐Roda *et al*., 2015). Considering the distinct phenotypes of flagella and *rhbE* mutants *in vitro* (Figs 2, 4) and *in planta* (Figs 3, 5), this is likely not the case in this strain. Thus, the observed *in planta* phenotype of *rhbE* is likely due to impairment of a flagella‐independent motility process.

The impact of impaired surface motility of the *rhbE* mutant on host infection likely did not arise from a defect in reaching root hairs, since the roots were flood‐inoculated similarly to the flagella mutants. It is possible that the *rhbE* mutant is impaired in attachment or spreading on the root surface, as seen in a previous study (Amaya‐Gómez *et al*., 2015). However, it has been shown that spreading on the root surface primarily occurs passively through root elongation, not bacterial motility (Caetano‐Anollés *et al*., 1988). Furthermore, no difference was observed in the number of ITs formed in root hairs by the *rhbE* mutant (Fig. 6b), suggesting that the symbiotic defects of *rhbE* are only manifested after the initiation of infection in our experimental system. The *rhbE* mutant strain strikingly promotes IT branching in root hairs (Fig. 6), raising the question of the need for Rhb1021 for normal IT development. It is possible that Rhb1021 secretion in the infection chamber somehow regulates the oriented tip growth of the IT. Recent cytochemical studies suggested ROS enrichment of the IT to possibly regulate cell wall stiffness during IT growth (Tsyganova *et al*., 2024). As siderophores have been associated with ROS sequestering or production (reviewed in Arnold, 2024), there may be a connection between Rhb1021 and ROS‐mediated cell wall modifications for IT growth. It would be interesting to investigate how an *rhbE* mutation would affect ROS homeostasis or other cellular mechanisms of IT growth (Jamet *et al*., 2003, 2007; Puppo *et al*., 2013; de Carvalho‐Niebel *et al*., 2024). Alternatively, the excessive branching phenotype observed in the *rhbE* mutant may be an indirect response by the plant to compensate for impaired nodule colonization. A number of infection‐defective plant mutants exhibit such a branching phenotype, which generally points to defective IT progression from root hairs to the cortex (Tansengco *et al*., 2003; Middleton *et al*., 2007; Gao *et al*., 2022). Investigating the spatiotemporal framework of rhizobactin production in rhizobia, using gene expression reporters, could help to understand its need during successive stages of IT formation and progression. Branched ITs suggest misdirected polar‐oriented growth. Using available plant *in vivo* markers (de Carvalho‐Niebel *et al*., 2024; Guillory *et al*., 2024) could help reveal potential modifications in IT polarity or cell wall interface in *rhbE* ITs.

Overall, our data suggest that *S. meliloti* uses flagella‐independent surface translocation through the secretion of the surfactant Rhb1021 to fine‐tune directed IT tip growth for optimal colonization of developing nodules of *M. truncatula*. Though *rhbE* mutation impacts proper nodule colonization and root hair IT development, ITs can still form and progress towards developing nodules. Thus, it is likely that rhizobia motility inside ITs depends not only on Rhb1021 secretion but also on other mechanisms. The galactoglucan exopolysaccharide (EPS II) is used by *S. meliloti* to promote surface motility (Nogales *et al*., 2012). However, *S. meliloti* 2011, the strain used in this work, has a mutation in the *expR* gene, which encodes a key activator of EPS II biosynthesis (Pellock *et al*., 2002). *S. meliloti* can also synthesize succinoglycan (EPS I) (Cheng & Walker, 1998) with major roles in early rhizobia infection signalling (Acosta‐jurado *et al*., 2021). Like other surfactants, EPS I biosynthesis can also promote surface motility in *S. meliloti*, though this has only been shown in overexpression conditions (Nogales *et al*., 2012). Future genetic or live microscopy studies may help elucidate the expression of EPS I biosynthesis genes during host infection and the potential involvement of this compound in rhizobia motility inside the IT.

Finally, slow motility requires surfactants but also may involve force generated from cell proliferation. Our model and live imaging in early IT formation suggest that the force generated by proliferation may represent an order of magnitude of *c*. 15% of all mechanisms responsible for the bacterial motion. This observation differs from Gage modeling and observations made with mature ITs, where sliding motility was suspected to be the main mechanism of motility. An investigation of the spatiotemporal dynamics of bacterial cell proliferation during host infection could therefore further expand our understanding of how rhizobia move through the IT.

## Competing interests

None declared.

## Author contributions

FdC‐N and A Becker conceived and supervised the study. JF generated confocal images for the modeling studies that were performed by FB and RP. A Bennion generated and validated the strains with the help of EK. A Bennion, AG, LF and EK performed the *in vitro* motility tests. AD did the mutant phenotypic studies in *Medicago* with the help of AG and LF. LM set up the slide scanning system for *in planta* infection quantification. JS mapped and analyzed mutant genomic data. AD, A Bennion, AG, LF, JS, JF, FC‐N and A Becker analyzed and interpreted the experimental data. AD, A Bennion and FdC‐N wrote the paper with inputs from A Becker, RP, JF and AG. AD and A Bennion contributed equally to this work.

## Disclaimer

The New Phytologist Foundation remains neutral with regard to jurisdictional claims in maps and in any institutional affiliations.

## Supporting information


**Fig. S1** Tracking bacteria within ITs.
**Fig. S2** Nodulation of *Sinorhizobium meliloti* flagella mutants in other conditions.
**Fig. S3** Nodulation of *Sinorhizobium meliloti rhtA* Rhizobactin 1021 transporter mutant.
**Table S1** Bacterial strains and plasmids.
**Table S2** Synthetic DNA used in this work.
**Table S3** Plasmid construction.
**Table S4** *Sinorhizobium meliloti* genome sequencing.
**Table S5** Bacterial motility modes.
**Table S6** *fliF–fliRdel* mutant genome sequence.
**Table S7** *rhbE* mutant genome sequence.
**Table S8** *rhtA* mutant genome sequence.
Please note: Wiley is not responsible for the content or functionality of any Supporting Information supplied by the authors. Any queries (other than missing material) should be directed to the *New Phytologist* Central Office.

## Data Availability

The authors declare that all data supporting the results of this study are available in the article and in Supporting Information. Genome sequence data are available in ArrayExpress under the accession no. E‐MTAB‐16345.

## References

[nph70897-bib-0001] Acosta‐jurado S , Fuentes‐romero F , Ruiz‐sainz JE , Janczarek M , Vinardell JM . 2021. Rhizobial exopolysaccharides: genetic regulation of their synthesis and relevance in symbiosis with legumes. International Journal of Molecular Sciences 22: 6233. 34207734 10.3390/ijms22126233 PMC8227245

[nph70897-bib-0002] Amaya‐Gómez CV , Hirsch AM , Soto MJ . 2015. Biofilm formation assessment in *Sinorhizobium meliloti* reveals interlinked control with surface motility. BMC Microbiology 15: 1–14. 25887945 10.1186/s12866-015-0390-z PMC4381460

[nph70897-bib-0003] Ames P , Bergman K . 1981. Competitive advantage provided by bacterial motility in the formation of nodules by *Rhizobium meliloti* . Journal of Bacteriology 148: 728–729. 7298580 10.1128/jb.148.2.728-729.1981 PMC216262

[nph70897-bib-0004] Arnold E . 2024. Non‐classical roles of bacterial siderophores in pathogenesis. Frontiers in Cellular and Infection Microbiology 14: 1–9. 10.3389/fcimb.2024.1465719 PMC11449898 39372500

[nph70897-bib-0005] Aroney STN , Pini F , Kessler C , Poole PS , Sánchez‐Cañizares C . 2024. The motility and chemosensory systems of *Rhizobium leguminosarum*, their role in symbiosis, and link to PTSNtr regulation. Environmental Microbiology 26: 1–15. 10.1111/1462-2920.16570 PMC7617929 38216524

[nph70897-bib-0006] Aroney STN , Poole PS , Sánchez‐Cañizares C . 2021. Rhizobial chemotaxis and motility systems at work in the soil. Frontiers in Plant Science 12: 1–15. 10.3389/fpls.2021.725338 PMC8429497 34512702

[nph70897-bib-0007] Bernabéu‐Roda L , Calatrava‐Morales N , Cuéllar V , Soto MJ . 2015. Characterization of surface motility in *Sinorhizobium meliloti*: regulation and role in symbiosis. Symbiosis 67: 79–90.

[nph70897-bib-0008] Bernabéu‐Roda L , López‐Ráez J , Soto MJ . 2021. Analyzing the effect of strigolactones on the motility behavior of rhizobia. Methods in Molecular Biology 2309: 93–103. 10.1007/978-1-0716-1429-7_8 34028681

[nph70897-bib-0009] Burch AY , Shimada BK , Mullin SWA , Dunlap CA , Bowman MJ , Lindow SE . 2012. *Pseudomonas syringae* coordinates production of a motility‐enabling surfactant with flagellar assembly. Journal of Bacteriology 194: 1287–1298. 22194459 10.1128/JB.06058-11 PMC3294827

[nph70897-bib-0010] Caetano‐Anollés G , Wall LG , De Micheli AT , Macchi EM , Bauer WD , Favelukes G . 1988. Role of motility and chemotaxis in efficiency of nodulation by *Rhizobium meliloti* . Plant Physiology 86: 1228–1235. 16666059 10.1104/pp.86.4.1228 PMC1054656

[nph70897-bib-0011] de Carvalho‐Niebel F , Fournier J , Becker A , Marín Arancibia M . 2024. Cellular insights into legume root infection by rhizobia. Current Opinion in Plant Biology 81: 102597. 39067084 10.1016/j.pbi.2024.102597

[nph70897-bib-0012] Catlow HY , Glenn AR , Dilworth MJ . 1990. The use of transposon‐induced non‐motile mutants in assessing the significance of motility of *Rhizobium leguminosarum* biovar *Trifolii* for movement in soils. Soil Biology and Biochemistry 22: 331–336.

[nph70897-bib-0013] Cerri MR , Frances L , Laloum T , Auriac MC , Niebel A , Oldroyd GED , Barker DG , Fournier J , de Carvalho‐Niebel F . 2012. *Medicago truncatula* ERN transcription factors: regulatory interplay with NSP1/NSP2 GRAS factors and expression dynamics throughout rhizobial infection. Plant Physiology 160: 2155–2172. 23077241 10.1104/pp.112.203190 PMC3510138

[nph70897-bib-0014] Cheng HP , Walker GC . 1998. Succinoglycan production by *Rhizobium meliloti* is regulated through the *ExoS‐ChvI* two‐component regulatory system. Journal of Bacteriology 180: 20–26. 9422587 10.1128/jb.180.1.20-26.1998 PMC106843

[nph70897-bib-0015] Compton KK , Scharf BE . 2021. Rhizobial chemoattractants, the taste and preferences of legume symbionts. Frontiers in Plant Science 12: 1–8. 10.3389/fpls.2021.686465 PMC8129513 34017351

[nph70897-bib-0016] Craig L , Forest KT , Maier B . 2019. Type IV pili: dynamics, biophysics and functional consequences. Nature Reviews Microbiology 17: 429–440. 30988511 10.1038/s41579-019-0195-4

[nph70897-bib-0017] Cuív PÓ , Clarke P , Lynch D , O'Connell M . 2004. Identification of *rhtX* and *fptX*, novel genes encoding proteins that show homology and function in the utilization of the siderophores rhizobactin 1021 by *Sinorhizobium meliloti* and pyochelin by *Pseudomonas aeruginosa*, respectively. Journal of Bacteriology 186: 2996–3005. 15126460 10.1128/JB.186.10.2996-3005.2004 PMC400637

[nph70897-bib-0018] Dilanji GE , Teplitski M , Hagen SJ , Hagen SJ . 2014. Entropy‐driven motility of *Sinorhizobium meliloti* on a semi‐solid surface. Proceedings of the Royal Society of London. Series B: Biological Sciences 281: 2013–2575. 10.1098/rspb.2013.2575 PMC4043075 24741008

[nph70897-bib-0019] Döhlemann J , Brennecke M , Becker A . 2016. Cloning‐free genome engineering in *Sinorhizobium meliloti* advances applications of *Cre/loxP* site‐specific recombination. Journal of Biotechnology 233: 160–170. 27393468 10.1016/j.jbiotec.2016.06.033

[nph70897-bib-0020] Esseling JJ , Lhuissier FGP , Emons AMC . 2003. Nod factor‐induced root hair curling: continuous polar growth towards the point of nod factor application. Plant Physiology 132: 1982–1988. 12913154 10.1104/pp.103.021634 PMC181283

[nph70897-bib-0021] Fournier J , Teillet A , Chabaud M , Ivanov S , Genre A , Limpens E , de Carvalho‐Niebel F , Barker DG . 2015. Remodeling of the infection chamber before infection thread formation reveals a two‐step mechanism for rhizobial entry into the host legume root hair. Plant Physiology 167: 1233–1242. 25659382 10.1104/pp.114.253302 PMC4378154

[nph70897-bib-0022] Fournier J , Timmers ACJ , Sieberer BJ , Jauneau A , Chabaud M , Barker DG . 2008. Mechanism of infection thread elongation in root hairs of *Medicago truncatula* and dynamic interplay with associated rhizobial colonization. Plant Physiology 148: 1985–1995. 18931145 10.1104/pp.108.125674 PMC2593660

[nph70897-bib-0023] Fujishige NA , Kapadia NN , De Hoff PL , Hirsch AM . 2006. Investigations of *Rhizobium* biofilm formation. FEMS Microbiology Ecology 56: 195–206. 16629750 10.1111/j.1574-6941.2005.00044.x

[nph70897-bib-0024] Gage DJ . 2002. Analysis of infection thread development using *Gfp*‐ and *DsRed*‐expressing *Sinorhizobium meliloti* . Journal of Bacteriology 184: 7042–7046. 12446653 10.1128/JB.184.24.7042-7046.2002 PMC135452

[nph70897-bib-0025] Gage DJ . 2004. Infection and invasion of roots by symbiotic, nitrogen‐fixing *Rhizobia* during nodulation of temperate legumes. Microbiology and Molecular Biology Reviews 68: 280–300. 15187185 10.1128/MMBR.68.2.280-300.2004 PMC419923

[nph70897-bib-0026] Gao J‐P , Jiang S , Su Y , Xu P , Wang J , Wenjie L , Cheng‐Wu L , Murray JD . 2022. Intracellular infection by symbiotic bacteria requires the mitotic kinase AURORA1. Proceedings of the National Academy of Sciences, USA 119: 1–11. 10.1073/pnas.2202606119 PMC9618073 36252014

[nph70897-bib-0027] Gao M , Coggin A , Yagnik K , Teplitski M . 2012. Role of specific quorum‐sensing signals in the regulation of exopolysaccharide II production within *Sinorhizobium meliloti* spreading colonies. PLoS ONE 7: 42611. 10.1371/journal.pone.0042611 PMC3418255 22912712

[nph70897-bib-0028] Gill PR , Barton LL , Scoble MD , Neilands JB . 1991. A high‐affinity iron transport system of *Rhizobium meliloti* may be required for efficient nitrogen fixation *in planta* . Plant and Soil 130: 211–217.

[nph70897-bib-0029] Gotz R , Schmitt R . 1987. *Rhizobium meliloti* swims by unidirectional, intermittent rotation of right‐handed flagellar helices. Journal of Bacteriology 169: 3146–3150. 3597320 10.1128/jb.169.7.3146-3150.1987 PMC212363

[nph70897-bib-0030] Guillory A , Fournier J , Kelner A , Hobecker K , Auriac MC , Frances L , Delers A , Pedinotti L , Le Ru A , Keller J *et al*. 2024. Annexin‐ and calcium‐regulated priming of legume root cells for endosymbiotic infection. Nature Communications 15: 1–21. 10.1038/s41467-024-55067-3 PMC11621553 39638784

[nph70897-bib-0031] Gurich N , González JE . 2009. Role of quorum sensing in *Sinorhizobium meliloti*‐alfalfa symbiosis. Journal of Bacteriology 191: 4372–4382. 19395488 10.1128/JB.00376-09 PMC2698488

[nph70897-bib-0033] Hoang HH , Becker A , González JE . 2004. The *LuxR* homolog *ExpR*, in combination with the *sin* quorum sensing system, plays a central role in *Sinorhizobium meliloti* gene expression. Journal of Bacteriology 186: 5460–5472. 15292148 10.1128/JB.186.16.5460-5472.2004 PMC490858

[nph70897-bib-0034] Holscher T , Kovacs AT . 2017. Sliding on the surface: bacterial spreading without an active motor. Environmental Microbiology 19: 2537–2545. 28370801 10.1111/1462-2920.13741

[nph70897-bib-0035] Huisman R , Geurts R . 2020. A roadmap toward engineered nitrogen‐fixing nodule symbiosis. Plant Communications 1: 100019. 33404552 10.1016/j.xplc.2019.100019 PMC7748023

[nph70897-bib-0036] Jamet A , Mandon K , Puppo A , Hérouart D . 2007. H_2_O_2_ is required for optimal establishment of the *Medicago sativa/Sinorhizobium meliloti* symbiosis. Journal of Bacteriology 189: 8741–8745. 17921312 10.1128/JB.01130-07 PMC2168964

[nph70897-bib-0037] Jamet A , Sigaud S , Van De Sype G , Puppo A , Hérouart D . 2003. Expression of the bacterial *catalase* genes during *Sinorhizobium meliloti–Medicago sativa* symbiosis and their crucial role during the infection process. Molecular Plant–Microbe Interactions 16: 217–225. 12650453 10.1094/MPMI.2003.16.3.217

[nph70897-bib-0038] Kawaharada Y , Nielsen MW , Kelly S , James EK , Andersen KR , Rasmussen SR , Füchtbauer W , Madsen LH , Heckmann AB , Radutoiu S *et al*. 2017. Differential regulation of the *Epr3* receptor coordinates membrane‐restricted rhizobial colonization of root nodule primordia. Nature Communications 8: 14534. 10.1038/ncomms14534 PMC5331223 28230048

[nph70897-bib-0039] Kearns DB . 2011. A field guide to bacterial swarming motility. Nature Reviews Microbiology 8: 634–644. 10.1038/nrmicro2405 PMC3135019 20694026

[nph70897-bib-0040] Krönauer C , Radutoiu S . 2021. Understanding Nod factor signalling paves the way for targeted engineering in legumes and non‐legumes. Current Opinion in Plant Biology 62: 102026. 33684882 10.1016/j.pbi.2021.102026

[nph70897-bib-0041] Leong SA , Williams PH , Ditta GS . 1985. Analysis of the 5′ regulatory region of the gene for 6‐aminolevulinic acid synthetase of *Rhizobium meliloti* . Nucleic Acids Research 13: 5965–5976. 2994020 10.1093/nar/13.16.5965 PMC321926

[nph70897-bib-0042] Lynch D , O'Brien J , Welch T , Clarke P , Cuív PO , Crosa JH , O'Connell M . 2001. Genetic organization of the region encoding regulation, biosynthesis, and transport of rhizobactin 1021, a siderophore produced by *Sinorhizobium meliloti* . Journal of Bacteriology 183: 2576–2585. 11274118 10.1128/JB.183.8.2576-2585.2001 PMC95175

[nph70897-bib-0043] Maier B , Wong GCL . 2015. How bacteria use type IV pili machinery on surfaces. Trends in Microbiology 23: 775–788. 26497940 10.1016/j.tim.2015.09.002

[nph70897-bib-0044] Malolepszy A , Kelly S , Sørensen KK , James EK , Kalisch C , Bozsoki Z , Panting M , Andersen SU , Sato S , Tao K *et al*. 2018. A plant chitinase controls cortical infection thread progression and nitrogen‐fixing symbiosis. eLife 7: 1–17. 10.7554/eLife.38874 PMC6192697 30284535

[nph70897-bib-0045] Mayjonade B , Gouzy J , Donnadieu C , Pouilly N , Marande W , Callot C , Langlade N , Muños S . 2016. Extraction of high‐molecular‐weight genomic DNA for long‐read sequencing of single molecules. BioTechniques 61: 203–205. 27712583 10.2144/000114460

[nph70897-bib-0046] McBride MJ . 2001. Bacterial gliding motility: multiple mechanisms for cell movement over surfaces. Annual Review of Microbiology 55: 49–75. 10.1146/annurev.micro.55.1.49 11544349

[nph70897-bib-0047] Mellor HY , Glenn AR , Arwas R , Dilworth MJ . 1987. Symbiotic and competitive properties of motility mutants of *Rhizobium trifolii TA1* . Archives of Microbiology 148: 34–39.

[nph70897-bib-0048] Middleton PH , Jakab J , Penmetsa RV , Starker CG , Doll J , Kaló P , Prabhu R , Marsh JF , Mitra RM , Kereszt A *et al*. 2007. An ERF transcription factor in *Medicago truncatula* that is essential for nod factor signal transduction. Plant Cell 19: 1221–1234. 17449807 10.1105/tpc.106.048264 PMC1913751

[nph70897-bib-0049] Miller LD , Yost CK , Hynes MF , Alexandre G . 2007. The major chemotaxis gene cluster of *Rhizobium leguminosarum* bv. *viciae* is essential for competitive nodulation. Molecular Microbiology 63: 348–362. 17163982 10.1111/j.1365-2958.2006.05515.x

[nph70897-bib-0050] Navarro‐Gómez P , Alías‐Villegas C , Jiménez‐Guerrero I , Fuentes‐Romero F , López‐Baena FJ , Acosta‐Jurado S , Vinardell JM . 2024. *Sinorhizobium fredii HH103 flgJ* is a flagellar gene induced by genistein in a *NodD1*‐ and *TtsI*‐dependent manner. Plant and Soil 505: 845–862.

[nph70897-bib-0051] Nogales J , Bernabéu‐Roda L , Cuéllar V , Soto MJ . 2012. *ExpR* is not required for swarming but promotes sliding in *Sinorhizobium meliloti* . Journal of Bacteriology 194: 2027–2035. 22328673 10.1128/JB.06524-11 PMC3318473

[nph70897-bib-0052] Nogales J , Domínguez‐Ferreras A , Amaya‐Gómez CV , van Dillewijn P , Cuéllar V , Sanjuán J , Olivares J , Soto MJ . 2010. Transcriptome profiling of a *Sinorhizobium meliloti fadD* mutant reveals the role of rhizobactin 1021 biosynthesis and regulation genes in the control of swarming. BMC Genomics 11: 157. 20210991 10.1186/1471-2164-11-157 PMC2848241

[nph70897-bib-0053] Ozer E , Yaniv K , Chetrit E , Boyarski A , Meijler MM , Berkovich R , Kushmaro A , Alfonta L . 2021. An inside look at a biofilm: *Pseudomonas aeruginosa* flagella biotracking. Science Advances 7: 1–15. 10.1126/sciadv.abg8581 PMC8195488 34117070

[nph70897-bib-0054] Pellock BJ , Teplitski M , Boinay RP , Bauer WD , Walker GC . 2002. A *LuxR* homolog controls production of symbiotically active extracellular polysaccharide II by *Sinorhizobium meliloti* . Journal of Bacteriology 184: 5067–5076. 12193623 10.1128/JB.184.18.5067-5076.2002 PMC135333

[nph70897-bib-0055] Puppo A , Pauly N , Boscari A , Mandon K , Brouquisse R . 2013. Hydrogen peroxide and nitric oxide: key regulators of the legume–*Rhizobium* and mycorrhizal symbioses. Antioxidants & Redox Signaling 18: 5136. 10.1089/ars.2012.5136 23249379

[nph70897-bib-0056] Salas ME , Lozano MJ , Lopez JL , Draghi WO , Serrania J , Torres Tejerizo GA , Albicoro FJ , Nilsson JF , Pistorio M , Del Papa MF *et al*. 2017. Specificity traits consistent with legume‐rhizobia coevolution displayed by *Ensifer meliloti* rhizosphere colonization. Environmental Microbiology 19: 3423–3438. 28618121 10.1111/1462-2920.13820

[nph70897-bib-0057] Sallet E , Roux B , Sauviac L , Jardinaud M‐F , Carrère S , Faraut T , de Carvalho‐Niebel F , Gouzy J , Gamas P , Capela D *et al*. 2013. Next‐generation annotation of prokaryotic genomes with EuGene‐P: application to *Sinorhizobium meliloti 2011* . DNA Research 20: 339–353. 23599422 10.1093/dnares/dst014 PMC3738161

[nph70897-bib-0058] Schäfer A , Tauch A , Jäger W , Kalinowski J , Thierbach G , Pühler A . 1994. Small mobilizable multi‐purpose cloning vectors derived from the. Gene 145: 69–73. 8045426 10.1016/0378-1119(94)90324-7

[nph70897-bib-0059] Schnabel E , Journet EP , De Carvalho‐Niebel F , Duc G , Frugoli J . 2005. The *Medicago truncatula SUNN* gene encodes a CLV1‐like leucine‐rich repeat receptor kinase that regulates nodule number and root length. Plant Molecular Biology 58: 809–822. 16240175 10.1007/s11103-005-8102-y

[nph70897-bib-0060] Soto MJ , Fernández‐Pascual M , Sanjuan J , Olivares J . 2002. A *fadD* mutant of *Sinorhizobium meliloti* shows multicellular swarming migration and is impaired in nodulation efficiency on alfalfa roots. Molecular Microbiology 43: 371–382. 11985715 10.1046/j.1365-2958.2002.02749.x

[nph70897-bib-0061] Sourjik V , Muschler P , Scharf B , Schmitt R . 2000. *VisN* and *VisR* are global regulators of chemotaxis, flagellar, and motility genes in *Sinorhizobium* (*Rhizobium*) *meliloti* . Journal of Bacteriology 182: 782–788. 10633114 10.1128/jb.182.3.782-788.2000 PMC94343

[nph70897-bib-0062] Sourjik V , Sterr W , Platzer J , Bos I , Haslbeck M , Schmitt R . 1998. Mapping of 41 chemotaxis, flagellar and motility genes to a single region of the *Sinorhizobium meliloti* chromosome. Gene 223: 283–290. 9858749 10.1016/s0378-1119(98)00160-7

[nph70897-bib-0063] Stevens JB , Carter RA , Hussain H , Carson KC , Dilworth MJ , Johnston AWB . 1999. The *fhu* genes of *Rhizobium leguminosarum*, specifying siderophore uptake proteins: *FhuDCB* are adjacent to a pseudogene version of *fhuA* . Microbiology 145: 593–601. 10217493 10.1099/13500872-145-3-593

[nph70897-bib-0064] Tansengco ML , Hayashi M , Kawaguchi M , Imaizumi‐anraku H . 2003. *crinkle*, a novel symbiotic mutant that affects the infection thread growth and alters the root hair, trichome, and seed development in *Lotus japonicus* . Plant Physiology 131: 1054–1063. 12644658 10.1104/pp.102.017020 PMC166871

[nph70897-bib-0065] Timofeeva AM , Galyamova MR , Sedykh SE . 2022. Bacterial siderophores: classification, biosynthesis, perspectives of use in agriculture. Plants 11: 3065. 36432794 10.3390/plants11223065 PMC9694258

[nph70897-bib-0066] Tsyganova AV , Gorshkov AP , Vorobiev MG , Tikhonovich IA , Brewin NJ , Tsyganov VE . 2024. Dynamics of hydrogen peroxide accumulation during tip growth of infection thread in nodules and cell differentiation in pea (*Pisum sativum* L.) symbiotic nodules. Plants 13: 2933. 39458872 10.3390/plants13202923 PMC11510766

[nph70897-bib-0067] Wadhwa N , Berg HC . 2022. Bacterial motility: machinery and mechanisms. Nature Reviews Microbiology 20: 161–173. 34548639 10.1038/s41579-021-00626-4

[nph70897-bib-0068] Wheatley RM , Ford BL , Li L , Aroney STN , Knights HE , Ledermann R , East AK , Ramachandran VK , Poole PS . 2020. Lifestyle adaptations of *Rhizobium* from rhizosphere to symbiosis. Proceedings of the National Academy of Sciences, USA 117: 23823–23834. 10.1073/pnas.2009094117 PMC7519234 32900931

[nph70897-bib-0069] Xiao TT , Schilderink S , Moling S , Deinum EE , Kondorosi E , Franssen H , Kulikova O , Niebel A , Bisseling T . 2014. Fate map of *Medicago truncatula* root nodules. Development 141: 3517–3528. 25183870 10.1242/dev.110775

[nph70897-bib-0070] Yang J , Lan L , Jin Y , Yu N , Wang D , Wang E . 2022. Mechanisms underlying legume–rhizobium symbioses. Journal of Integrative Plant Biology 64: 244–267. 34962095 10.1111/jipb.13207

[nph70897-bib-0071] Zatakia HM , Nelson CE , Syed UJ , Scharf BE . 2014. *ExpR* coordinates the expression of symbiotically important, bundle‐forming *Flp* Pili with quorum sensing in *Sinorhizobium meliloti* . Applied and Environmental Microbiology 80: 2429–2439. 24509921 10.1128/AEM.04088-13 PMC3993167

[nph70897-bib-0072] Zheng H , Mao Y , Teng J , Zhu Q , Ling J , Zhong Z . 2015. Flagellar‐dependent motility in *Mesorhizobium tianshanense* is involved in the early stage of plant host interaction: study of an *flgE* mutant. Current Microbiology 70: 219–227. 25287045 10.1007/s00284-014-0701-x

