# Supplementary material for: Rhizobial motility preference in root colonization of Medicago truncatula
Source: New Phytol. 2026 Jan 15;250(1):547–62. doi: 10.1111/nph.70897 (PMC12961261; doi:10.1111/nph.70897)
Supplement: Supplementary file 1 — Fig. S1 Tracking bacteria within ITs. Fig. S2 Nodulation of Sinorhizobium meliloti flagella mutants in other conditions. Fig. S3 Nodulation of Sinorhizobium meliloti rhtA Rhizobactin 1021 transporter mutant. Table S1 Bacterial strains and plasmids. Table S2 Synthetic DNA used in this work. Table S3 Plasmid construction. Table S4 Sinorhizobium meliloti genome sequencing. Table S5 Bacterial motility modes. Table S6 fliF–fliRdel mutant genome sequence. Table S7 rhbE mutant genome sequence. Table S8 rhtA mutant genome sequence. Please note: Wiley is not responsible for the content or functionality of any Supporting Information supplied by the authors. Any queries (other than missing material) should be directed to the New Phytologist Central Office. [file NPH-250-547-s001.pdf]

New Phytologist

***New Phytologist* Supporting Information**

Article title: **Rhizobial motility preference in root colonization of *Medicago truncatula***

Authors: Anaïs Delers[1§], Anne Bennion[1,2,3§], Ambre Guillory[1], Lisa Frances[1], Elizaveta Krol[2,3], Fanny Bonnafous[4], Laurena Medioni[5], Javier Serrania[2], Rémi Peyraud[4], Joëlle Fournier[1], Fernanda de Carvalho-Niebel*[1] and Anke Becker*[2,3].

Article acceptance date: 15 December 2025

The following Supporting Information is available for this article:

Fig. S1 Tracking bacteria within ITs.

Fig. S2 Nodulation of *S. meliloti* flagella mutants in other conditions.

Fig. S3 Nodulation of *S. meliloti rhtA* Rhizobactin 1021 transporter mutant.

Table S1 Bacterial strains and plasmids.

Table S2 Synthetic DNA used in this work.

Table S3 Plasmid construction.

Table S4 *S. meliloti* genome sequencing.

Table S5 Bacterial motility modes.

Table S6 *fliF-fliRdel* mutant genome sequence.

Table S7 *rhbE* mutant genome sequence.

Table S8 *rhtA* mutant genome sequence.

Fig. S1 Tracking *S. meliloti* within ITs. Regions of interest (ROIs) corresponding to individual bacteria (magenta) were created using the MicrobeJ plugin of ImageJ. Each rhizobial cell in the file was numbered starting from the IT tip (B1, B2, etc), at time point 1. Bacteria that had divided during a time interval (B3, B5, B10, B11) were named after the parent cell. The ROIs corresponding to the infection chamber (IC) and a bacterium close to it (Bx) were used as spatial references. When individual bacteria could not be distinguished (see B7 and B8 at time point 2) or a bacterium divided over the next time interval (see B3, for example), their speed was not measured (and is thus missing in Fig. **1b**). Spatial coordinates of each ROI were determined and exported using MicrobeJ.

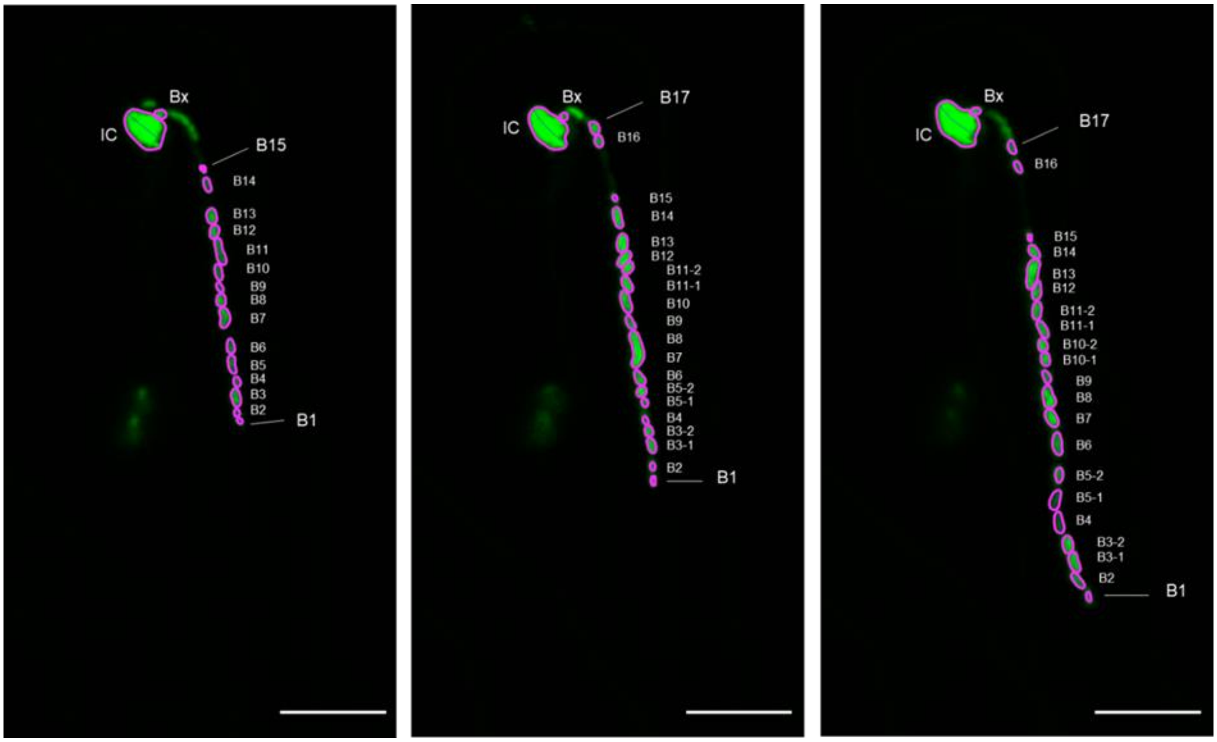

Fig. S2 *S. meliloti fliF* and *fliF-fliRdel* flagella motility mutants form nodules fully colonized by rhizobia in *Medicago truncatula* in different growth conditions.

M. truncatula plants grown in inert attapulgite substrate were inoculated with lacZ-expressing *S. meliloti* WT or *fliF* and *fliF-fliRdel* mutant strains. Number of nodules (Nod) and nodule primordia (NP) per plant (**a**) and of infected Nod/NP per plant (**b**) were quantified in nodulated roots using a binocular magnifying glass at 7 dpi (days post inoculation) (WT n = 36; *fliF* n = 36; *fliF-fliRdel* n = 36). Box plots (**a**-**b**) show the distribution of values (circles) from 3 independent experiments. First and third quartiles (horizontal box edges), minimum and maximum (whisker tips), median (centerline), mean (solid black triangle), and outliers (crosses) are shown. Ns (non-significant) indicate no statistical difference relative to WT (p = 0,894 in **a**, p = 0,799 in **b**; one-way ANOVA). (**c**) Representative images of LacZ-stained roots of *M. truncatula* at 7 dpi with WT and mutant *S. meliloti* strains. Fully-infected nodules (arrowheads) are observed in both WT and mutant-inoculated plants. Scale bars in (**c**) = 3 mm.

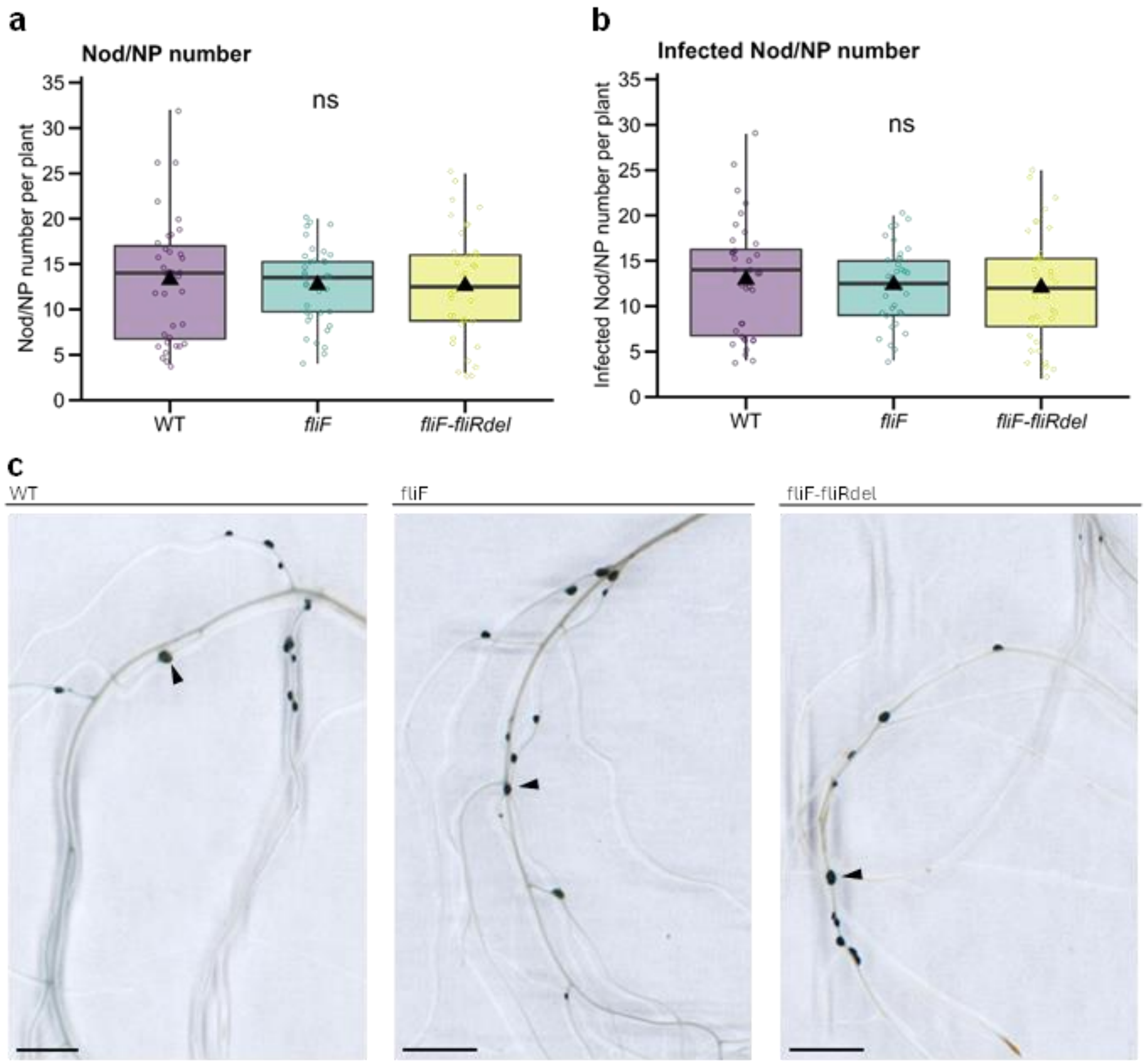

New Phytologist

Fig. S3 Mutation in *rhtA* Rhizobactin 1021 transporter does not affect early rhizobia nodule colonization. *M. truncatula* A17 grown in vermiculite: sand pots were inoculated with *S. meliloti* WT, *rhtA,* and *rhbE* mutant strains, and quantification of Nod/NP infection level (X-gal staining intensity) was performed at 7 dpi (days post inoculation) in individual Nod/NP (WT n = 352; *rhtA* n = 342; *rhbE* n = 357). Box plots show the distribution of values (circles) from 2 independent experiments. First and third quartiles (horizontal box edges), minimum and maximum (whisker tips), median (centerline), mean (solid black triangle), and outliers (crosses) are shown. Letters indicate statistically significant differences between groups (p = 6.276046e-06, Kruskal-Wallis α = 5%).

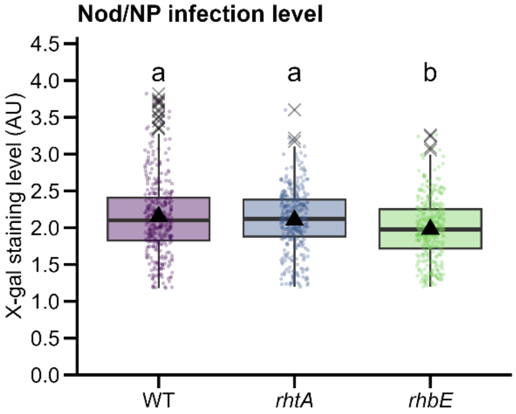

Table S1 Bacterial strains and plasmids.

**Strains or plasmids**	**Properties**	**Reference**
E. coli strains		
DH5α	*E. coli, recA1*, Δ*lacU169*, Δ80d*lacZ*, ΔM15	Bethesda research Laboratories
XL1-Blue pRK2013	*E. coli* XL-1 blue MRF', *recA1 lac thi* [F' traD36 *proAB lacI* ^{q} *Z*ΔM15] Tc^r	Bullock *et al.,* 1987
S17-I	*E. coli* 294, *thi*, RP4-2-Tc::Mu-Km::Tn*7* integrated into the chromosome	Simon, 1984
DH5α pAB14	DH5α with pAB14 plasmid Spec^r	This work
S17-I pXLGD4	S17-I with pXLGD4 plasmid, Tc^r	This work
S17-I pAB42	S17-I with pAB42 plasmid, Tc^r	This work
S17-I pAB43	S17-I with pAB43 plasmid, Tc^r	This work
S17-I pAB44	S17-I with pAB44 plasmid, Tc^r	This work
S. meliloti strains		
*Sm*2011 WT	*S. meliloti* 2011 wild type, *expR*$^-$, Strep^r	Casse *et al.*, 1979
*Sm*2011-lacZ	*Sm*2011 WT with pXLBD4 plasmid, Strep^r Tc^r	Cerri *et al.*, 2012
*Sm*2011-GFP	*Sm*2011 WT with pHc60 plasmid, Tc^r	Fournier *et al.*, 2015
*Sm*2011-mScarlet	*Sm*2011 WT with pAB14 plasmid, Strep^r Spec^r	This work
Δ*fliF*	*Sm*2011 with a markerless deletion of *fliF* gene (*SMc03014*) (chr<711589 – 713262>), Strep^r	H. Wendt, SYNMIKRO
Δ*fliF*-lacZ	Δ*fliF* with pXLGD4 plasmid Strep^r Tc^r	This work
*fliF-fliR*del	*Sm*2011 with markerless deletion of ~40 kb from *fliF* (*SMc03014*) to *fliR* (SMc03055) (chr<711589 – 751436>), Strep^r	This work
*fliF-fliR*del-lacZ	*fliF-fliR*del with pXLGD4 plasmid Strep^r Tc^r	This work
Δ*rhbE*	*Sm*2011 with a markerless deletion of *rhbE* gene (MpA<1311399 – 1312769>), Strep^r	This work
Δ*rhbE*-lacZ	Δ*rhbE* with pXLGD4 plasmid Strep^r Tc^r	This work
Δ*rhbE*-mScarlet	Δ*rhbE* with pAB14 plasmid Strep^r Spec^r	This work
Δ*rhtA*	*Sm*2011 with a markerless deletion of *rhtA* gene, (MpA<1315745 – 1317985>) Strep^r	This work
Δ*rhtA*-lacZ	*rhtA* with pXLGD4 plasmid Strep^r Tc^r	This work
Plasmids		
pABC-Psyn	Mobilizable repABC-based mini-replicon, single-copy in *S. meliloti*, Spec r	Schäper *et al.*, 2019
pK18mobsacB	Suicide vector, pUC18 derivate, *lacZ*α, *mob* site, *sacB,* Km^r	Schäfer *et al.*, 1994
pXLGD4	pRK290 derivative, *hemA*(*SMc03104*)::*lacZ*, Tc^r	Leong *et al.*, 1985
pAB14	pABC-psyn-p*SMc06412::mScarlet-I,* Spec^r	This work
pAB42	pK18mobsacB-*fliF* 5'UTR$_{-1565 – -1}$::*fliR* 3'UTR$_{1 – 1565}$, Km^r	This work
pAB43	pK18mobsacB-p*rbhE*$_{-709 – -1}$::*rhbE*::*rhbE* 3'UTR$_{1 – 714}$, Km^r	This work
pAB44	pK18mobsacB-p*rhtA*$_{-700 – -1}$::*rhbA*::*rhbA* 3'UTR$_{1 – 698}$	This work

New Phytologist

Table S2 Synthetic DNA used in this work.

ID	Name	Sequence
1	mScarlet coding sequence<br><br>Original sequence (Bindels *et al.* 2016) codon optimized for *S. meliloti* and synthesized by Twist Bioscience	ATGGTCAGCAAGGGCGAAGCCGTCATCAAAGAATTCATGCGCTTCAAGGTCCAC<br>ATGGAAGGCTCGATGAACGGCCACGAGTTCGAGATCGAAGGCGAAGGCGAAG<br>GCCGGCCGTATGAGGGCACCCAGACCGCCAAGCTCAAGGTCACCAAAGGCGGC<br>CCGCTGCCGTTCTCGTGGGACATCCTTTCGCCGCAGTTCATGTATGGCTCGCGCG<br>CATTCATCAAGCATCCGGCAGACATCCCGGACTATTATAAGCAGTCGTTCCCGGAA<br>GGCTTCAAGTGGGAGCGCGTCATGAACTTCGAGGATGGCGGCGCAGTCACCGT<br>CACGCAGGACACCTCGCTTGAGGACGGCACCCTCATCTATAAGGTCAAGCTTCGC<br>GGCACGAACTTCCCGCCGGATGGCCCGGTCATGCAGAAAAAGACCATGGGCTG<br>GGAAGCCTCGACCGAGCGGCTCTATCCGGAAGATGGCGTCCTGAAGGGCGACA<br>TCAAGATGGCCCTCCGCCTCAAAGATGGCGGCCGCTACCTCGCCGACTTCAAGA<br>CCACCTATAAGGCCAAGAAACCGGTCCAGATGCCGGGCGCATATAACGTCGACC<br>GCAAGCTCGACATCACCTCGCACAACGAGGACTATACCGTCGTCGAGCAGTATGA<br>GCGCTCGGAAGGCCGCCATTCGACCGGCGGCATGGACGAGCTGTATAAGTGA
2	PncA-f	ttaagcggccgcAAATGCAAAAAAAGCAGATGACAGCGCCCAAGGTTTGAGCTAATT<br>TCGGCGCAAAGTGTCGTTGTGGAAAAGATTCCGATAGGAGAAAGAAATGgctagc<br>atat
3	PncA-r	atatgctagcCATTTCTTTCTCCTATCGGAATCTTTTCCACAACGACACTTTGCGCCGA<br>AATTAGCTCAAACCTTGGGCGCTGTCATCTGCTTTTTTTGCATTTgcggccgcttaa
4	mScarlet-MCS-f	atagcggccgcaagcttagatctatagctagcATGGTCAGCAAGGGCGAGGC
5	mScarlettSm-Kpn-r	ataGGTACCTCACTTATACAGCTCGTCCATG
6	rhbE-US-f	atagaattcaTGCAAGGCGAATCTCAAG
7	rhbE-US-r	ataggtaccCATAATCCGAATTGTTGAATG
8	rhbE-DS-f	ataggtaccCTCATGCATCATGATCCGC
9	rhbE-DS-r	atatctagaTGGATGCACCGGCATCATG
10	rhtA-US-f	atatctagacggCATGTTCCTCGCTCTGA
11	rhtA-US-r	ataggatcccatAACGCCCCCTGCGCAC
12	rhtA-DS-f	ataggatccGACCGGGGTGATCGCGGAC
13	rhtA-DS-r	tctaagcttGGATCGGCGAACATTCGCTC
14	flaXX-HR1-XbaI-f	atatctagaTATGCCCTGACAGGCGTCGAG
15	flaXX-HR1-NotI-r	atatgcggccgcTCTGTTTCCGCACCCAGTAT
16	flaXX-HR2-NotI-f	atatagcggccgcGCTCGATGAAGGCGCGGT
17	flaXX-HR2-HindIII-r	gcagaagcttCAGAAATCGCTGTAGCGGAA

For primers, annealing regions are indicated with uppercase letters, and appendix sequences added for cloning purposes are indicated with lowercase letters.

Table S3 Plasmid construction.

Plasmid	Construction process
pAB14	A 50 bp region of the *SMc06412* promoter and a 44 bp *mucR* leader and Shine Dalgarno sequence and its reverse complement (oligonucleotides 2 and 3, Table **S2**) were hybridized and digested with NotI and NheI. The mScarlet coding sequence (oligonucleotide 1, Table **S2**) was PCR-amplified (oligonucleotides 4 and 5, Table **S2**) and digested with NheI and KpnI. Inserts were ligated into the pABC-Psyn vector opened with NotI and KpnI and dephosphorylated with FastAP to prevent recyclization.
pAB42	A ~1,5 kb region upstream of *fliF* was PCR-amplified from Rm2011 genomic DNA (oligonucleotides 14 and 15, Table S4) and digested with XbaI and NotI. Likewise, a ~1,5 kb region downstream of *fliR* was amplified (oligonucleotides 16 and 17, Table **S2**) and digested with NotI and HindIII. These inserts were ligated into the pK18mobsacB vector opened with XbaI and HindIII and dephosphorylated with FastAP to prevent recyclization.
pAB43	A ~700 bp region upstream of *rhbE* was PCR-amplified from Rm2011 genomic DNA upstream (oligonucleotides 6 and 7), digested with EcoRI and Acc65I. Likewise, a ~700 bp region downstream of *rhbE* was amplified (oligonucleotides 8 and 9) and digested with Acc65I and XbaI. These inserts were ligated into the pK18mobsacB vector opened with EcoRI and XbaI and dephosphorylated with FastAP to prevent recyclization.
pAB44	A ~700 bp region upstream of *rhtA* was PCR-amplified from Rm2011 genomic DNA (oligonucleotides 10 and 11) and digested with XbaI and BamHI. Likewise, a ~700 bp region downstream of *rhtA* was amplified (oligonucleotides 12 and 13) and digested with BamHI and HindIII. These inserts were ligated into the pK18mobsacB vector opened with XbaI and HindIII and dephosphorylated with FastAP to prevent recyclization.

Table S4 *S. meliloti* genome sequencing.

Strain	Sequencing method	Total bp sequenced	Total reads	Raw coverage	Assembled coverage
2011 WT (parental strain)	ONT	961.039.771	62.828	138 x	>10 x
fliF-fliRdel	ONT	879.718.826	60.341	126 x	>10 x
rhbE	ONT	425.882.923	110.059	63 x	62 x
rhtA	ONT	270.334.017	60.801	40 x	39 x

Table S5 Bacterial motility modes.

	Velocity	
**Motility mode**	**Direction**	**Speed**
Swimming	straight	5 mm h^{-1}
Swarming	straight	10 mm h^{-1}
Surfing	straight	1 mm h^{-1}
Twitching	Jerky	1 mm h^{-1}
Gliding	jerky	90 µm h^{-1}
Sliding	straight	Growth rate x colony size

The different modes of motility employed by bacteria and their associated speed and direction.

New Phytologist

Table S6 Comparison of genome sequences of the *fliF-fliRdel* mutant to that of the parental *S. meliloti* WT and *Sm*2011 (GMI11495).

Replicon	Consensus Position	Type	Length	Consensus	Allele	Count	Coverage	Frequency	Forward/reverse balance	Average quality	Overlapping annotations	Coding region change	Amino acid change
chromosome	1716200	Insertion	1	-	G	92	106	86.79245283	0.467391304	29.07608696			
chromosome	1716251	Insertion	7	-	CGGCCTG	102	106	96.22641509	0.441176471	41.10364146			
chromosome	2059077	Insertion	42	-	GGCGGCGGCGGCGGCGGCGGTGGCGGCGGTGGCGGCGGTGGC	75	82	91.46341463	0.493333333	35.73111111	CDS: SMc04232	SMc04232:c.358_359insGTGGCGGCGGTGGCGGCGGTGGCGGCGGCGGCGGCGGCGGCG	SMc04232: p.Gly119_Asp120insGlyGlyGlyGlyGlyGlyGlyGlyGlyGlyGlyGlyGlyGly
chromosome	3622706	SNV	1	T	C	137	141	97.16312057	0.467153285	38.87591241			
chromosome	3622726	MNV	2	AA	GG	137	141	97.16312057	0.481751825	40.19708029			
chromosome	3622729	Deletion	1	G	-	136	141	96.45390071	0.485294118	39.91176471			
chromosome	3622746	SNV	1	A	G	138	141	97.87234043	0.471014493	38.21014493			
chromosome	3650478	SNV	1	G	A	119	123	96.74796748	0.470588235	41.6302521	CDS: SMc02799-rsmG	SMc02799-rsmG:c.151C>T	SMc02799-rsmG: p.His51Tyr
pSymB	726015	Insertion	3	-	CAG	94	103	91.26213592	0.457446809	40.19858156	CDS: SMb21087-traA2	SMb21087-traA2:c.33_35dupCAG	SMb21087-traA2: p.Ile11_Arg12insSer
pSymB	726064	SNV	1	G	A	101	103	98.05825243	0.455445545	44.37623762	CDS: SMb21087-traA2	SMb21087-traA2:c.82G>A	SMb21087-traA2: p.Ala28Thr
pSymB	726067	SNV	1	A	C	101	103	98.05825243	0.475247525	43.69306931	CDS: SMb21087-traA2	SMb21087-traA2:c.85A>C	
pSymB	726090	SNV	1	A	C	103	103	100	0.466019417	45.19417476	CDS: SMb21087-traA2	SMb21087-traA2:c.108A>C	
pSymB	726105	SNV	1	C	T	103	103	100	0.466019417	46.44660194	CDS: SMb21087-traA2	SMb21087-traA2:c.123C>T	
pSymB	726120	SNV	1	C	G	103	103	100	0.466019417	38.84466019	CDS: SMb21087-traA2	SMb21087-traA2:c.138C>G	
pSymB	726123	SNV	1	A	G	102	103	99.02912621	0.460784314	41.81372549	CDS: SMb21087-traA2	SMb21087-traA2:c.141A>G	
pSymB	727959	SNV	1	C	T	107	107	100	0.46728972	44.54205607	CDS: SMb21087-traA2	SMb21087-traA2:c.1977C>T	
pSymB	729484	SNV	1	T	C	98	109	89.90825688	0.397959184	20.56122449	CDS: SMb21087-traA2	SMb21087-traA2:c.3502T>C	
pSymB	729508	SNV	1	G	A	101	110	91.81818182	0.405940594	42.26732673	CDS: SMb21087-traA2	SMb21087-traA2:c.3526G>A	SMb21087-traA2:p.Glu1176Lys
pSymB	729543	MNV	2	CA	TG	110	110	100	0.436363636	43.70454545	CDS: SMb21087-traA2	SMb21087-traA2:c.3561_3562delinsTG	SMb21087-traA2:p.Thr1188Ala

pSymB	729548	SNV	1	A	T	109	110	99.09090909	0.431192661	44.40366972	CDS: SMb21087-traA2	SMb21087-traA2:c.3566A>T	SMb21087-traA2:p.Tyr1189Phe
pSymA	413296	SNV	1	G	T	117	120	97.5	0.401709402	39.82051282	CDS: SMa0763	SMa0763:c.198G>T	SMa0763 (hypothetical protein): p.Leu66Phe
pSymA	568951	Insertion	1	-	A	119	123	96.74796748	0.462184874	41.89915966			
pSymA	711591	Deletion	39847								fliF to fliR		
pSymA	911589	SNV	1	A	G	100	102	98.03921569	0.48	38.17			
pSymA	1183827	SNV	1	G	T	125	125	100	0.416	46.68	CDS: SMa2101	SMa2101:c.992G>T	SMa2101: p.Gly331Val

Legend

Variation detected both in the *flif-fliR*del mutant and the parental WT when compared to Sm2011 (GMI11495)

Deletion of the *fliF* to *fliR* region (*fliF-fliR*del)

Variation not detected in the parental WT, but in the *flif-fliR*del, *rhbE*, and *rhtA* mutants, indicating that it occurred before mutant constructions

Variation only found in the *flif-fliR*del mutant

New Phytologist

Table S7 Comparison of genome sequences of the *rhbE* mutant to that of the parental *S. meliloti* WT and *Sm*2011 (GMI11495).

Mapping	Consensus Position	Type	Length	Consensus	Allele	Count	Coverage	Frequency	Forward/reverse balance	Average quality	Overlapping annotations	Coding region change	Amino acid change
chromosome	1716200	Insertion	1	-	G	48	59	81.3559322	0.35416667	28.2083333			
chromosome	1716251	Insertion	7	-	CGGCCTG	54	59	91.5254237	0.42592593	38.8624339			
chromosome	2059077	Insertion	42	-	GGCGGCGGCGGCGGCGGCGGTGGCGGCGGTGGCGGCGGTGGC	32	38	84.2105263	0.46875	39.4501488	CDS: SMc04232	SMc04232:c.358_359insGTGGCGGCGGTGGCGGCGGTGGCGGCGGCGGCGGCGGCGGCG	SMc04232:p.Gly119_Asp120insGlyGlyGlyGlyGlyGlyGlyGlyGlyGlyGlyGlyGlyGly
chromosome	3622706	SNV	1	T	C	87	88	98.8636364	0.48275862	34.6206897			
chromosome	3622726	MNV	2	AA	GG	89	89	100	0.48314607	37.3764045			
chromosome	3622729	Deletion	1	G	-	88	89	98.8764045	0.48863636	36.5909091			
chromosome	3622746	SNV	1	A	G	90	90	100	0.48888889	35.3222222			
chromosome	3650478	SNV	1	G	A	88	92	95.6521739	0.47727273	42.1590909	CDS: SMc02799-rsmG	SMc02799-rsmG:c.151C>T	SMc02799-rsmG:p.His51Tyr
pSymB	726015	Insertion	3	-	CAG	51	59	86.440678	0.47058824	37.4313725	CDS: SMb21087-traA2	SMb21087-traA2:c.33_35dupCAG	SMb21087-traA2:p.Ile11_Arg12insSer
pSymB	726064	SNV	1	G	A	59	59	100	0.44067797	40.1186441	CDS: SMb21087-traA2	SMb21087-traA2:c.82G>A	SMb21087-traA2:p.Ala28Thr
pSymB	726067	SNV	1	A	C	59	59	100	0.44067797	38.9661017	CDS: SMb21087-traA2	SMb21087-traA2:c.85A>C	
pSymB	726090	SNV	1	A	C	59	60	98.3333333	0.45762712	42.9830508	CDS: SMb21087-traA2	SMb21087-traA2:c.108A>C	
pSymB	726105	SNV	1	C	T	62	62	100	0.4516129	41.8548387	CDS: SMb21087-traA2	SMb21087-traA2:c.123C>T	
pSymB	726120	SNV	1	C	G	62	62	100	0.4516129	35.1612903	CDS: SMb21087-traA2	SMb21087-traA2:c.138C>G	
pSymB	726123	SNV	1	A	G	61	62	98.3870968	0.45901639	37.8360656	CDS: SMb21087-traA2	SMb21087-traA2:c.141A>G	

pSymB	727959	SNV	1	C	T	55	57	96.4912281	0.45454545	38.4909091	CDS: SMb21087-traA2	SMb21087-traA2:c.1977C>T	
pSymB	729484	SNV	1	T	C	57	62	91.9354839	0.40350877	18.754386	CDS: SMb21087-traA2	SMb21087-traA2:c.3502T>C	
pSymB	729508	SNV	1	G	A	60	62	96.7741935	0.41666667	39.75	CDS: SMb21087-traA2	SMb21087-traA2:c.3526G>A	SMb21087-traA2:p.Glu1176Lys
pSymB	729543	MNV	2	CA	TG	62	62	100	0.43548387	41.9919355	CDS: SMb21087-traA2	SMb21087-traA2:c.3561_3562delinsTG	SMb21087-traA2:p.Thr1188Ala
pSymB	729548	SNV	1	A	T	61	62	98.3870968	0.42622951	42.0655738	CDS: SMb21087-traA2	SMb21087-traA2:c.3566A>T	SMb21087-traA2:p.Tyr1189Phe
pSymB	838025	Deletion	1	G	-	45	53	84.9056604	0.44444444	13.4	CDS: SMb21264	SMb21264:c.881delG	SMb21264 (putative glycosyl transferase): p.Gly294fs
pSymA	568951	Insertion	1	-	A	56	59	94.9152542	0.5	38.7321429			
pSymA	616106	Deletion	1	T	-	70	71	98.5915493	0.47142857	34.1571429			
pSymA	911589	SNV	1	A	G	47	50	94	0.40425532	37.7446809			
pSymA	1183827	SNV	1	G	T	60	60	100	0.45	40.8666667	CDS: SMa2101	SMa2101:c.992G>T	SMa2101:p.Gly331Val
pSymA	1302927	Deletion	2	TC	-	61	77	79.2207792	0.49180328	31.4918033			
pSymA	1309739	Deletion	1354	ATCATGACGGATTTCGATCTGGCAGGGATCGGCATCGGGCCGTTCAACCTTGGCCTTGCCGCCCTTCTCTCTTCCCACGAAAATCTCTCGAACGTGTTTCTCGAGCGCAAACCGGCGTTCCGGTGGCACGAAGGGCTCATTCTTCCCGGGACGACGCTGCAGGTTCCCTTCATGGCGGATCTCGTCACCATGGCGGACCCGACGCATCGTTTGAGCTTCCTCAACTACCTTGCTGTGCACGATCGCCTCTACAAGTTCTACTTCTACGAAAACTTCATGATCCCGCGGCAGGAATACGATCACTATTGCCGCTGGGCCTCACAGCAACTTTCGGCGTGCCGCTTCGGCGAGGAAGTGGTCGACGTCGCCCATGAGTCGGCATCCGACAGCTTCATTGTCGAAAGCAGGTCCGCCTCGGGCGGAAAGCAGCAATATCGAAGCCGTAATATTGCGATCGGCGTCGGCACGGCTCCTTT	-	48	50	96	0.5	34.3125	CDS: SMa2406-rhbD, CDS: SMa2408-rhbE	SMa2406-rhbD:c.591_*1340del; SMa2408-rhbE:c.4_1357del	SMa2406-rhbD:p.*197fs; SMa2408-rhbE:p.Ile2_Ser448del

				CCTGCCGAAGTGGGCGCAGATCAAGACGTTGGCACCGCTCATGCATTCCAGCGAATTCGGGAGGCGGCGTCTGGAGCTTTCGAAACGACGCCGTGTAACAGTCATCGGCTCAGGGCAAAGCGCTGCCGAATGCGTTCTTGCGCTTTTGAACGATCTGACGCCGGAGATGGTAGCAGCCGGCGCTTCGATCCAGTGGATCACCCGATCGGCGGGGTTCTTTCCGATGGAGTATTCGAAGCTGGGCCTCGAATACTTCACGCCTGACTACATGCGCCACTTCCATCGAATCGCGCCCGTTAGACGCCGCGAGATCGTTGCAGACCAGGGCCTGCTCTACAAGGGCATCAGCTTTTCGACGATCGGTGAGATCTTCGATCTCATGTATGAACGTTCGGTGGGCGGACGCGATCCAGGCCTAGCACTCTTTTCCAATTGTGCGGTCGAAACGCTGGAGAGCGCAGGGGGATCGGGGTCGTTTCGCATCGGCATCAACCACAACCATCTGGATGAGAAGGCAACGGTCGAGACCGACGCGATTGTGGCCGCAACGGGGTACCGGCACGCGTGGCCGGAATGGCTGGGTTCGCTGAAGGGCAGCGTCCTCGATACCTGCGAGTGGGGCGACCTCGTCGTCGGCGGGGATTTCCGTGCACGCCGCAGCGACGGCGGCAAGGGCCATGTGTTCGTCCAGAATGCCGAAACCTTCCACCACGGCGTCGGCGCGCCGGATCTTGGCCTCGGCGCCTTTCGCAATGCCGTCATCGTCAATCAGCTTCTCGGTCGCGAGCATTACCGCGTCAATGCGTCGGCATCGTTCCAGAAGTTCGGTCTGCCCTCCAGCCAGACCGCTCCGTCCTCGATTTCA									

Legend

Variation detected both in the *rhbE* mutant and the parental WT when compared to Sm2011 (GMI11495)

Deletion of *rhbE* (includes deletion of the third base of the stop codon of the upstream *rhbD* gene; a stop codon is reconstituted by fusion to the 3'-end of deletion resulting in a functional *rhbD* cds)

Variation not detected in the parental WT, but in the *flif-fliR*del, *rhbE*, and *rhtA* mutants, indicating that it occurred before mutant constructions

Variation only found in the *rhbE* mutant

New Phytologist

Table S8 Comparison of genome sequences of the *rhtA* mutant to that of the parental *S. meliloti* WT and *Sm*2011 (GMI11495).

Mapping	Consensus Position	Type	Length	Consensus	Allele	Count	Coverage	Frequency	Forward/reverse balance	Average quality	Overlapping annotations	Coding region change	Amino acid change
chromosome	1716200	Insertion	1	-	G	38	43	88.372093	0.5	26.2105263			
chromosome	1716251	Insertion	7	-	CGGCCTG	40	43	93.0232558	0.475	39.7428571			
chromosome	2059077	Insertion	42	-	GGCGGCGGCGGCGGCGGCGGTGGCGGCGGTGGCGGCGGTGGC	15	19	78.9473684	0.266666667	37.8428571	CDS: SMc04232	SMc04232:c.358_359insGTGGCGGCGGTGGCGGCGGTGGCGGCGGCGGCGGCGGCG	SMc04232:p.Gly119_Asp120insGlyGlyGlyGlyGlyGlyGlyGlyGlyGlyGlyGlyGlyGly
chromosome	3622706	SNV	1	T	C	46	48	95.8333333	0.47826087	35.4782609			
chromosome	3622726	MNV	2	AA	GG	47	47	100	0.489361702	38.6808511			
chromosome	3622729	Deletion	1	G	-	47	47	100	0.489361702	37.2765957			
chromosome	3622746	SNV	1	A	G	46	47	97.8723404	0.47826087	36.0869565			
chromosome	3650478	SNV	1	G	A	40	44	90.9090909	0.45	42.75	CDS: SMc02799-rsmG	SMc02799-rsmG:c.151C>T	SMc02799-rsmG:p.His51Tyr
pSymB	726015	Insertion	3	-	CAG	31	35	88.5714286	0.419354839	38.0322581	CDS: SMb21087-traA2	SMb21087-traA2:c.33_35dup CAG	SMb21087-traA2:p.Ile11_Arg12insSer
pSymB	726064	SNV	1	G	A	34	34	100	0.441176471	41.7941176	CDS: SMb21087-traA2	SMb21087-traA2:c.82G>A	SMb21087-traA2:p.Ala28Thr
pSymB	726067	SNV	1	A	C	34	34	100	0.441176471	40.1764706	CDS: SMb21087-traA2	SMb21087-traA2:c.85A>C	
pSymB	726090	SNV	1	A	C	32	32	100	0.4375	44.25	CDS: SMb21087-traA2	SMb21087-traA2:c.108A>C	
pSymB	726105	SNV	1	C	T	31	32	96.875	0.419354839	44.6451613	CDS: SMb21087-traA2	SMb21087-traA2:c.123C>T	

pSymB	726120	SNV	1	C	G	31	31	100	0.451612903	36.3548387	CDS: SMb21087-traA2	SMb21087-traA2:c.138C>G	
pSymB	726123	SNV	1	A	G	31	31	100	0.451612903	38.5806452	CDS: SMb21087-traA2	SMb21087-traA2:c.141A>G	
pSymB	727959	SNV	1	C	T	37	38	97.3684211	0.486486486	39.7567568	CDS: SMb21087-traA2	SMb21087-traA2:c.1977C>T	
pSymB	729484	SNV	1	T	C	30	31	96.7741935	0.366666667	18.2333333	CDS: SMb21087-traA2	SMb21087-traA2:c.3502T>C	
pSymB	729508	SNV	1	G	A	30	31	96.7741935	0.366666667	37.9	CDS: SMb21087-traA2	SMb21087-traA2:c.3526G>A	SMb21087-traA2:p.Glu1176Lys
pSymB	729543	MNV	2	CA	TG	31	31	100	0.387096774	43.8548387	CDS: SMb21087-traA2	SMb21087-traA2:c.3561_3562delinsTG	SMb21087-traA2:p.Thr1188Ala
pSymB	729548	SNV	1	A	T	31	31	100	0.387096774	40.8387097	CDS: SMb21087-traA2	SMb21087-traA2:c.3566A>T	SMb21087-traA2:p.Tyr1189Phe
pSymA	568951	Insertion	1	-	A	42	44	95.4545455	0.476190476	38.3809524			
pSymA	911589	SNV	1	A	G	30	30	100	0.466666667	34.7			
pSymA	1183827	SNV	1	G	T	34	34	100	0.5	42.7941176	CDS: SMa2101	SMa2101:c.992G>T	SMa2101:p.Gly331Val
pSymA	1314087	Deletion	2235	CAACAATGAGAATGGCGGCATAAGCTTTTGCGTCTTCGTCGTCGTAATTGGATTTGGCACGGGTGCTGTTGCGCAGGAGCCAGCGAATCAATCCGAAGCTGTGACGAGCCTGGAAGAAATCGTAGTCACCGGCGGACGATCCGCTCAGCAGATTTCTGAAATCGCGCGCACCATTTACGTCGTCGATTCGGATCAGATCCAGGCCGAGGCCCGGTCGGGCAAGACGCTGCAGCAGATTCTGGGCGAAACCATTCCGAGCTTCGACCCCGCCAGCGACGGAGCGCGTACGTCATTCGGCCAGAACCTGCGTGGCCGGCCACCACTGATCCTTGTCGACGGTGTCTCGATGAACTCGGCGCGCTCGCTGAGCCGACAATTCGATGCCATCGATCCATTCAACATCGAACGGGTGGAGGTCCTGTCTGGGGCAACTGCAATCTACGGCGGGAACGCAACCGGCGGCATC	-	33	36	91.6666667	0.454545455	31.5151515	CDS: SMa2414-rhtA	SMa2414-rhtA:c.6_2240del	SMa2414-rhtA:p.Asn3_*747del

				ATCAACATCATCACGAAGAAGGGC<br>AAGGACGCGGAACCGGGCCTGCAT<br>GCGGAAGTGACCGGCGGCATGGGC<br>AGCGGCTTTGCCGGCAGCCAGGAC<br>TTCGACCGCAATGCGGCGGGTGCG<br>GTCACCTATAACAGCGAAAACTGG<br>GATGCCCGCCTTTCGATCGCCGGCA<br>ACAGGACCGGCGCCTTTTATGACGG<br>CAGCGGCACGCTGCTGATCCCCGAC<br>ATCACGCAGACGTCCACCGCATTCA<br>ACGAGCGCATCGACCTGATGGGGT<br>CTATCGGCTACCAGATCGACGACGA<br>CCGTCGCGTCGAATTTTCCGGGCAA<br>TATTTCGACAGCAAGCAGGACTCGG<br>ATTACGGGCTTTATTACGGGCCCTT<br>CTTTGCAGCGCTCGCCGACCCGAGC<br>CTGTTTGAAACCCGTTCCGGATACG<br>AGTCCGACTTCAATCCGCAGACACG<br>CCGCTCGATGTTGAACGTCACCTAT<br>ACCGATAATGACGTTTTCGGTCAGC<br>AGCTATTGCTTCAGGGATCTTACCG<br>GACTGAGCGGATAAAGTTCCATCCC<br>TTCCCTGCTTCGGGCAATAGCGAAA<br>CGGGTCCTTACTTTTACGGCAGTTC<br>GCAGGACACAGACTATTACGGCATC<br>AGGGCTGCCTTGGTTGCGGAACCC<br>ACCGACGCGCTGAAAATCACCTATG<br>GCATCGATGCGGATATGGATTCCTT<br>CACGGCGCGTCAGAACATCTTCGAC<br>ATGGTGGCAGCCGGGCAATCCGGG<br>GGGCTCGATTTCAATACGATCGGCA<br>AGACCGGTCTTTATCCATCCATCGA<br>TGTCTCCACCGTTGCCGGCTTTGCT<br>GAGGCCTCGTACGAGGCTACCGAC<br>AGGCTCACTCTGAACGGCGGGGTG<br>CGCTATCAGTTCGTCAACACTGAGG<br>TATCCGACTTCATCGGAGCGGCTCA<br>GCAGGTCGCCATCCTGCAGGGCAG<br>AGCAACGTCTGCCGACACCATACCT<br>GGCGGCGAAGTCAATTACGACGCC<br>GCTTTGTTCAGTGCAGGCGCAACCT<br>ACCAGTTGACGAATACGCAGCAGG<br>TCTACGCCAATTTCAGCCAGGGCTT<br>CGAACTACCAGATCCGGCCAAGTAC<br>TATGGAATTGGCAACTACTCCTTCTC<br>CGGTGGACACTACACGCTTGTCAAT<br>AGCGTGAACGTGGGCGATTCCGCG									

				CTCGAGGCGATCAAGACCAACTCAT TCGAAATCGGCTATCGTCTCGATGA CGGCACCTTCAATCTTGAAACGGCG GCCTACTACTCGCTCTCCGATCGTTC CATCAATCTCAATCGCTCATCGCTTG CCGTTGAGATCATCGATCGGGAGA GACGCGTCTATGGCATCGAAGGAA AGGCGGGCGTAAAGCTCGATCATG GCTTCGATGTTGGAGTTCTGGGGCA CTGGGTCAGAACCGAGGTCAAGGG AGCCGACGGTTGGGAAAAGGACTC CGTCGGCAGCGCGAGCGTCTCCAA ACTCGGCGGCTATGTCGGCTGGACC AATGACGCTCTCAGCCTGAGGTTTT CCGGTCAGCACATCTTCGAACTCAC CGACGCCCAGAATTTCACGATCGAC GATTATACGTTGTTTGATCTAACCG GCGGCTACAGGTTCGAGAATACCG ACACGACGCTGAATTTCGGTATCCA TAACGTCTTCGATACTGATTACACC ACCGTCTGGGGCTCTCGCGCCAAAG CCCTTTACGGCGGCCTAGCCGACGA GTCGGTGTTCGACTACAAGGGCCG CGGCCGCACGTTCGCGGTCTCGCTG ACAAAGGTTTTTA										

Legend

Variation detected both in the *rhtA* mutant and the parental WT when compared to Sm2011 (GMI11495)

Deletion of *rhtA*

Variation not detected in the parental WT, but in the *flif-fliR*del, *rhbE*, and *rhtA* mutants, indicating that it occurred before mutant constructions

References

Bullock W, Fernandez JM and Short JM. 1987. XL1-Blue—a high-efficiency plasmid transforming *recA Escherichia coli* strain with β-galactosidase selection. *Biotechniques* G: 376-379.

Casse F, Boucher C, Julliot JS. **1979**. Identification and characterization of large plasmids in *Rhizobium meliloti* using agarose Gel electrophoresis. *Journal of General Microbiology* **113**: 229–242.

Cerri MR, Frances L, Laloum T, Auriac MC, Niebel A, Oldroyd GED, Barker DG, Fournier J, de Carvalho-Niebel F. **2012**. *Medicago truncatula* ERN transcription factors: Regulatory interplay with NSP1/NSP2 GRAS factors and expression dynamics throughout rhizobial infection. *Plant Physiology* **160**: 2155–2172.

Fournier J, Teillet A, Chabaud M, Ivanov S, Genre A, Limpens E, Carvalho-Niebel F De, Barker DG. **2015**. Remodeling of the infection chamber before infection thread formation reveals a two-step mechanism for rhizobial entry into the host legume root hair. *Plant Physiology* **167**: 1233–1242.

Leong SA, Williams PH, Ditta GS. **1985**. Analysis of the 5' regulatory region of the gene for 6-aminolevulinic acid synthetase of *Rhizobium meliloti*. *Nucleic Acids Research* **13**: 5965–5976.

Schäfer A, Tauch A, Jäger W, Kalinowski J, Thierbach G, Pühler A. **1994**. Small mobilizable multi-purpose cloning vectors derived from the. *Gene* **145**: 69–73.

Schäper S, Wendt H, Bamberger J, Sieber V, Schmid J, Becker A. **2019**. A Bifunctional UDP-Sugar 4-Epimerase Supports Biosynthesis of Multiple Cell Surface Polysaccharides in *Sinorhizobium meliloti*. **201**: 1–18.

Simon R. **1984**. High frequency mobilization of gram-negative bacterial replicons by the in vitro constructed *Tn 5-Mob* transposon. *MGG Molecular & General Genetics* **196**: 413–420.

New Phytologist
